# Genome-Wide Mapping of the Escherichia coli PhoB Regulon Reveals Many Transcriptionally Inert, Intragenic Binding Sites

**DOI:** 10.1128/mbio.02535-22

**Published:** 2023-04-17

**Authors:** Devon M. Fitzgerald, Anne M. Stringer, Carol Smith, Pascal Lapierre, Joseph T. Wade

**Affiliations:** a Wadsworth Center, New York State Department of Health, Albany, New York, USA; b Department of Biomedical Sciences, School of Public Health, University at Albany, Albany, New York, USA; Massachusetts Institute of Technology

**Keywords:** ChIP-seq, PhoB, pho regulon, transcription factors

## Abstract

Genome-scale analyses have revealed many transcription factor binding sites within, rather than upstream of, genes, raising questions as to the function of these binding sites. Here, we use complementary approaches to map the regulon of the Escherichia coli transcription factor PhoB, a response regulator that controls transcription of genes involved in phosphate homeostasis. Strikingly, the majority of PhoB binding sites are located within genes, but these intragenic sites are not associated with detectable transcription regulation and are not evolutionarily conserved. Many intragenic PhoB sites are located in regions bound by H-NS, likely due to shared sequence preferences of PhoB and H-NS. However, these PhoB binding sites are not associated with transcription regulation even in the absence of H-NS. We propose that for many transcription factors, including PhoB, binding sites not associated with promoter sequences are transcriptionally inert and hence are tolerated as genomic “noise.”

## INTRODUCTION

Bacteria encode numerous transcription factors (TFs) that regulate transcription initiation by binding DNA near promoters and modulating the ability of RNA polymerase (RNAP) holoenzyme to bind promoter DNA or to isomerize to an actively transcribing conformation (1). TF function has been studied almost exclusively in the context of TF binding sites in intergenic regions, upstream of the regulated genes. However, genome-scale analyses of TF binding have identified large numbers of intragenic binding sites, far from gene starts. The proportion of binding sites for a TF that are intragenic varies extensively between different TFs (2, 3), with some TFs having the majority of their binding sites inside genes (3–6). Despite the large number of intragenic TF binding sites, relatively little is known about their function.

Regulatory activity has been described for few intragenic TF binding sites and can be classified into the following distinct classes based on the regulatory target and mechanism of action: (i) canonical regulation of transcription initiation of the downstream gene, generating an RNA with an extended 5′ untranslated region (UTR) that overlaps a gene (5, 7–10); (ii) canonical regulation of transcription initiation of a stable noncoding RNA that initiates inside a gene or 3′ UTR (11, 12); (iii) regulation of transcription initiation of the gene that contains the TF binding site (mechanisms of regulation in almost all such cases are unknown [3], although transcription repression can occur from a site close to the promoter due to a physical interaction with a more upstream site, resulting in formation of a DNA loop [13, 14]); and (iv) regulation of transcription elongation due to the TF acting as a roadblock for RNAP (15–18). Another possible regulatory function for intragenic TF binding sites is the regulation of pervasive transcription—transcription of large numbers of short, unstable RNAs from inside genes, a process that is ubiquitous in bacteria (19, 20). Although there are no described examples of TFs that regulate unstable, intragenic transcripts, many of these RNAs are differentially expressed between growth conditions (21), consistent with regulation by TFs. Intragenic TF binding sites might also have functions that are not directly connected to gene regulation, such as facilitating short- or long-range chromosome contacts (22–25) or serving as TF-titrating decoy sites (26, 27). Lastly, it is possible that intragenic TF binding sites serve no biological function and arise as a consequence of genetic drift (28) or genome evolution that is constrained by selection for particular codons.

### PhoB is a conserved transcription factor that regulates phosphate homeostasis.

PhoB is a member of the PhoB/OmpR family of response regulator TFs and is a key regulator of phosphate homeostasis in many Gram-negative bacteria (29, 30). PhoB forms a two-component system with the sensor kinase PhoR (31). When inorganic phosphate (P_i_) levels are low, PhoR autophosphorylates and then phosphorylates PhoB (30, 31), triggering PhoB dimerization and DNA binding activity (30). Phosphorylated PhoB binds direct repeat sequences called *pho* boxes (32) and is a dual regulator, capable of both activating and repressing transcription depending on the position of the binding site.

In Escherichia coli and related species, PhoB regulates the expression of genes encoding the high-affinity phosphate transport system (*pst*), a phosphonate transport complex (*phn*), and the glycerol-3-phosphate transporter (*ugp*) and other genes related to phosphate homeostasis (30, 33). These genes are collectively referred to as the *pho* regulon. PhoB has been implicated in the regulation of a number of other cellular processes and stress responses, including motility, biofilm formation, quorum sensing, cell surface remodeling, the stringent response, and the general stress response (34, 35). Indeed, transcriptomic and proteomic studies of phosphate-depleted E. coli have suggested that the *pho* regulon has many additional members (36, 37). However, most of these putative regulon members have limited experimental support (30, 33).

Here, we describe a high-resolution, genome-wide mapping of the *pho* regulon using chromatin immunoprecipitation sequencing (ChIP-seq) and transcriptome sequencing (RNA-seq). We refine and expand the set of known *pho* regulon genes and identify many intragenic PhoB binding sites. We show that the large majority of intragenic PhoB binding sites are not conserved and are not associated with detectable regulatory function. Thus, our data suggest that individual intragenic PhoB sites are nonfunctional and that TFs can bind many intragenic sites with little or no impact on local transcription.

## RESULTS

### Genome-wide binding of PhoB under phosphate-limiting conditions.

**FIG 1:**
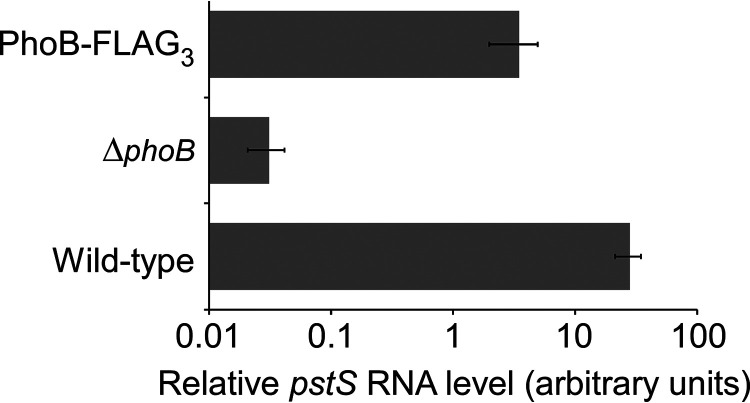
Partially reduced activity of the C-terminally FLAG_3_-tagged PhoB. qRT-PCR was used to measure levels of the *pstS* RNA relative to the *minD* RNA control in wild-type MG1655/pBAD24 (wild type), MG1655 Δ*phoB* (CDS091)/pBAD24 (Δ*phoB*), or MG1655 *phoB*-FLAG_3_ (DMF34)/pBAD24 (PhoB-FLAG_3_) for cells grown under low-phosphate conditions. Values are the average of three independent biological replicates; error bars represent ±1 standard deviation.

ChIP-seq is used to map the genome-wide binding of TFs. To facilitate ChIP-seq of E. coli PhoB, we introduced C-terminal FLAG tags at the native *phoB* locus. We used quantitative reverse transcriptase PCR (qRT-PCR) to measure the expression of *pstS*, a PhoB-activated gene, in wild-type cells, Δ*phoB* cells, and cells expressing *phoB*-FLAG_3_. Cells were grown in minimal medium with low phosphate levels to induce the kinase activity of PhoR. As expected, we observed a large decrease (~900-fold) in *pstS* levels in Δ*phoB* cells relative to those of wild-type cells (Fig. 1). In cells expressing PhoB-FLAG_3_, we observed a much smaller decrease (~8-fold) in *pstS* levels relative to those of wild-type cells (Fig. 1), indicating that the tagged PhoB derivative retains partial function.

**FIG 2:**
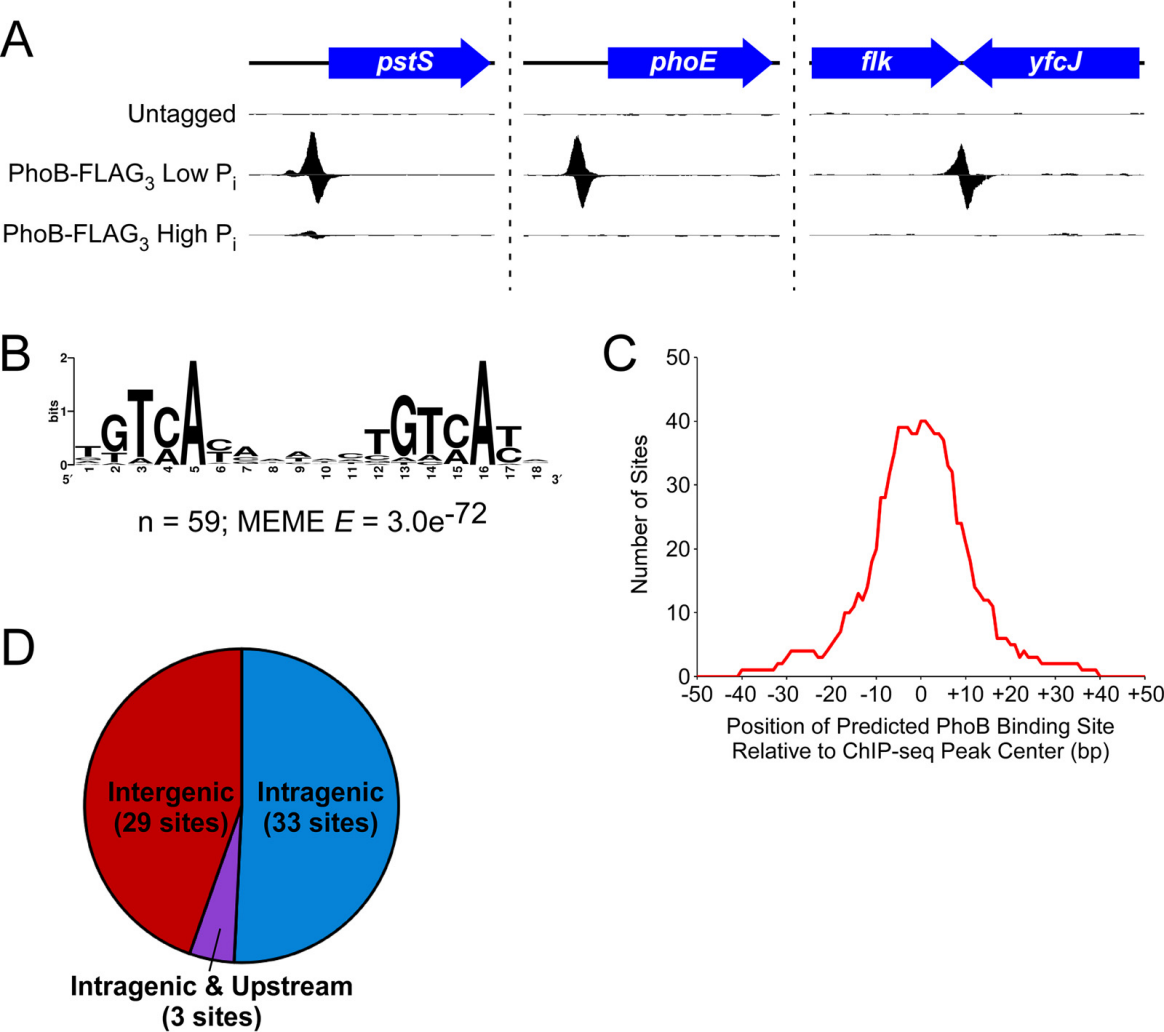
ChIP-seq identifies PhoB binding sites. (A) ChIP-seq data for (i) an untagged control under low-phosphate conditions, (ii) PhoB-FLAG_3_ under low-phosphate conditions, and (iii) PhoB-FLAG_3_ under high-phosphate conditions. Three genomic regions are shown, with one data set from two independent biological replicates. Values on the *x* axis represent genome positions. Values on the *y* axis represent normalized sequence read coverage, with positive values indicating sequence reads mapping to the forward strand and negative values indicating sequence reads mapping to the reverse strand. *y* axis scales differ between the three genomic regions but are matched for the three data sets for any given genomic region. (B) Significantly enriched DNA sequence motif derived from 100-bp regions surrounding each ChIP-seq peak. The number of sites contributing to the motif and the E value determined by MEME are indicated. (C) Analysis of the position of inferred PhoB binding sites relative to the position of ChIP-seq peak centers. For each of the binding sites contributing to the motif determined by MEME (see panel B), we determined the position of the binding site relative to the associated ChIP-seq peak center. The *x* axis indicates positions relative to ChIP-seq peak centers. The *y* axis indicates the number of binding sites that cover any given position. (D) Pie chart showing the genome context of PhoB binding sites identified by ChIP-seq. Sites designated “intragenic and upstream” are intragenic but <200 bp upstream of an annotated gene start.

We used ChIP-seq to map the genome-wide binding of PhoB-FLAG_3_ during growth under low-phosphate conditions. Thus, we identified 65 enriched regions (Fig. 2A; Table 1). For a control, we performed ChIP-seq with an untagged strain grown under the same conditions; none of the regions enriched in the PhoB-FLAG_3_ ChIP-seq data set were enriched in the control data set (Fig. 2A). We conclude that the 65 enriched regions in the PhoB-FLAG_3_ ChIP-seq data set are likely to represent genuine PhoB-bound regions. Note that a single PhoB-bound region could include more than one PhoB site, as is the case for the region upstream of *phoB* itself, which has been reported to include two PhoB sites (38). We identified a highly enriched sequence motif, with instances of the motif found in 59 of the 65 putative PhoB-bound regions (MEME E value = 3.0e^−72^) (Fig. 2B). This motif contains a clearly distinguishable direct repeat and is similar to the previously reported *pho* box consensus sequence (39). Furthermore, the identified motif is centrally enriched relative to the calculated ChIP-seq peak centers (Fig. 2C) (CentriMo E value = 1.2e^−12^). The presence and central enrichment of this motif at ChIP-seq peaks further support the veracity of PhoB-bound regions and confirm the high spatial resolution of the ChIP-seq data.

**TABLE 1:** List of PhoB-bound regions identified by ChIP-seq

Genome coordinate^*a*^	H-NS-bound?^*b*^	ChIP score^*c*^	Associated gene(s)^*d*^	Binding site sequence^*e*^	Expression wt^*f*^	Expression Δ*phoB*^*f*^^,^^*g*^
14763		1	(*dnaJ*)	NA	(8,326)	(4,689)
22367		2	*ileS*	TGTAATCAAACCGAAATA	21,580	24,295
201079		1	(*lpxD*)	GGTCACATTACGTTCATG	(9,261)	(11,591)
**260277**	×	14	***phoE***/*proB*	TGTAATAAAAGCGTAAAC	68,155/6,306	69*/4,330
262258		1	(*proA*)	TGTAATACGGTTGAAACG	(9,578)	(6,248)
**332492**	×	3	(*yahA*)	TGTAACAGAAATATCACA	(2,246)	(1,084*)
**401676**	×	20	***phoA***	**TGTCATAAAGTTGTCACG**	310,112	1,548*
**417093**		11	*sbcD*/***phoB***	**TTTCATAAATCTGTCATA**	274/32,731	271/34*
**417245**		5	(***phoB***)	ATTCACAGCACTGTCATA	(32,731)	(34*)
569907	×	6	(*ybcK*)	TGTCACATCGATGTAATC	(33)	(24)
**595521**		3	*cusR*/*cusC*	TGTCATTTTTCTGTCACC	391/208	239/50*
922786		2	*cspD*/*clpS*	TGTCACATTCCTGTCAAT	2,330/18,217	1,603/23,285
983795		2	(*gloC*)	TGTCAGGCCGCTGTCATC	(4,405)	(7,533*)
1015596		7	*rmf*	ATTCACGCCACTGTCATA	3,816	5,371
1065537	×	2	*agp*	AGTCATATTTCTGTCACA	976	1,983*
1084845		11	***phoH***	TGTCATCACTCTGTCATC	37,076	13,375*
1096396	×	1	(*ycdU*)	TGTCACAAAAGAATCACT	(53)	(64)
1117391		1	(*yceA*)	GATAAAAAAATCGTCATG	(560)	(456)
**1371360**	×	5	(*ycjM*)	GGTCACATTTATTTCATA	(11)	(14)
**1447326**		2	*feaR*/*feaB*	TTTCACAGAGCGAAAACG	111/786	379*/1,231
1527862	×	1	*rhsE*	TATCAGAAAAATGTCATG	ND	ND
**1577158**	×	4	(*yddB*)	CGGCACAAAACTGTCATA	(94)	(99)
**1581949**	×	2	(*ydeN*)	**TGTCAAAAATCAGTAATG**	(120)	(119)
1861798		5	(*yeaC*)	NA	(1,355)	(1,480)
1874218		1	*yoaI*/*yeaL*	TGTCATCAAACTGCCATT	12,266/36	28*/67
1948542		1	(*nudB*)	CGTCACGCCGCTGCAACA	(3,654)	(3,763)
**1978157**		1	(*flhD*)	**TGACATCAACTTGTCATA**	(71)	(57)
2055017		1	*amn*	TTTCACATTTCTGTGACA	42,299	12,429*
**2137862**	×	5	*wza*/*yegH*	TGTCACAATTCGATCATG	10/2,257	15/2,498
**2240543**		6	*mglB*	TGTAACCCGTATGTAACA	1,076	772
2438961		8	(*yfcJ*)	TGTCACGATACTGTCATT	(163)	(187)
2484480	×	2	(*evgS*)	AGTAACAACCGTGTCACA	(1,514)	(3,343*)
**2743442**		3	(*yfiN*) *yfiB*	**TGACACAAATCTTTAATC**	(575) 1,293	(656) 1,629
2799166		1	(*alaE*)	AGTCACGCTTGCGTCATG	(83)	(58)
2819248		1	*csrA*	TGTAATGTGTTTGTCATT	6,539	7,460
2976292		4	*omrA*	GGTCATCAATCTGTAACA	1	3
**3031605**		3	(*uacT*)	**CGTCACATTATTGCAATG**	(5)	(4)
**3081080**		2	(*tktA*)	**AGAAATACCGTTGTCATC**	(26,875)	(27,000)
3198259		1	(*glnE*)	TGTCATCTTCCTGCAACG	(11,291)	(9,028)
**3243474**		3	(*uxaC*) *uxaA*	**TGTCATACACCCGTCACG**	(608) 243	(465) 228
3309385		1	(*pnp*)	CTTCACAGTACCGTCATC	(33,309)	(53,983*)
**3362763**	×	4	(*yhcA*) *yhcD*	**GGTAATAAATATGTCACT**	(38) 425	(54) 415
3458489		1	(*gspE*)	NA	(69)	(51)
3573580		5	*glgB*	GGTCAAAAAAATGTCACA	21,445	17,405
**3592453**		35	***ugpB***	TGTCATCTTTCTGACACC	54,461	2,830*
3600942		2	*rpoH*	TTTCATCTCTATGTCACA	7,346	4,943
3648534		2	(*arsR*)	GGTAACAGAAATGACATA	(61)	(66)
**3672318**	×	2	*yhjB*/*yhjC*	**TTTCACAATGTTGTCATG**	252/397	218/408
3682037		1	*yhjJ*	GAACATGAAAATGTCACG	1,871	2,721
3709177		1	(*eptB*)	GGTCACCGAGTTGTCATA	(585)	(1,126*)
3728905		1	(*xylB*)	AGTAATCTTTCGGTAATA	(1,160)	(836)
3731396	×	1	(*xylF*)	NA	(46)	(31)
3767949	×	2	(*rhsJ*)	TTACATACAAATGTAATA	(ND)	(ND)
3776578		6	*yibT*/*yibL*	TGTAATAGTTCTGTAACG	5,264/1,129	2,601*/2,135*
**3911600**		38	***pstS***	**TGTCATAAAACTGTCATA**	239,097	310*
**3937206**		3	*rbsK*	TGTCACCATCAGGTCATA	1,452	1,701
3990899		1	*hemC*/*cyaA*	TTTCACGCCGTTGTAATA	3,823/16,054	5,169/18,141
4113746	×	1	(*fpr*)	CGTAAATGTTTCGTCATC	(4,069)	(4,643)
4128495		2	*metJ*/*metB*	NA	543/1,432	524/1,263
**4142314**		1	*frwA*/*frwC*	TGTAATGTAACCGTCAAT	72/35	60/36
**4227508**		1	(*metH*)	**ATTCACAAATCTGTCACT**	(18,026)	(25,993)
4245063		1	(*malF*)	TGTCATTAAAAAGAAACA	(486)	(853*)
**4325211**		2	***phnC***	**ATTAACCAAATCGTCACA**	32,470	13*
4459250		1	(*pmbA*)	GGTAACGATATTGAAACA	(11,462)	(11,004)
4522523	×	1	(*yjhG*)	NA	(478)	(444)

a Position of the center of the PhoB-bound region in the E. coli MG1655 genome (GenBank accession no. NC_000913.3). Positions in bold indicate those that overlap regions found by ChIP-chip previously (43).

b An “×” indicates that the region was previously reported to be bound by H-NS (49).

c A measure of relative enrichment from ChIP-seq data.

d Genes in parentheses indicate an intragenic binding site. Downstream genes are listed if the PhoB ChIP-seq peak center is in an intergenic region upstream of that gene and/or if the peak center is <200 bp upstream of the gene start. Previously described *pho* regulon members are in bold.

e Binding sites identified by MEME. Bold sequences match binding sites described previously (43). “NA” indicates ChIP-seq peaks for which no binding site sequence was found by MEME.

f Relative RNA levels in MG1655/pBAD24 (wild type [wt]) or MG1655 Δ*phoB* (CDS091)/pBAD24 (Δ*phoB*) for genes indicated in the column headed “Associated genes.” (See footnote *d* for explanation of parentheses.) “ND” indicates genes for which RNA levels were not determined.

g Asterisks indicate expression differences between the wild-type and Δ*phoB* strains that were determined to be statistically significant (*q *< 0.01).

The 65 PhoB-bound regions identified by ChIP-seq include most well-established PhoB sites, as well as many novel targets (Table 1). We identified PhoB-bound regions upstream of 7 of the 10 genes/operons described previously as being in the *pho* regulon (Table 1, underlined gene names) (33), with no ChIP-seq signal upstream of *waaH*, *ytfK*, or *psiE*. We also identified PhoB-bound regions upstream of the predicted *pho* regulon gene *amn* (Table 1) (40) and upstream of *yoaI*, which was described as a direct PhoB target in E. coli O157:H7 (41, 42). We identified 21 PhoB-bound regions upstream of genes/operons not previously described as part of the *pho* regulon and lacking a clear connection to phosphate homeostasis. The remaining 36 PhoB-bound regions, over half of the total sites identified by ChIP-seq, are located inside genes (Fig. 2D; Table 1). Strikingly, all but 3 intragenic PhoB binding sites are far from neighboring gene starts (>200 bp) and thus are unlikely to participate in promoter-proximal regulation of these genes (Fig. 2D; Table 1).

**FIG 3:**
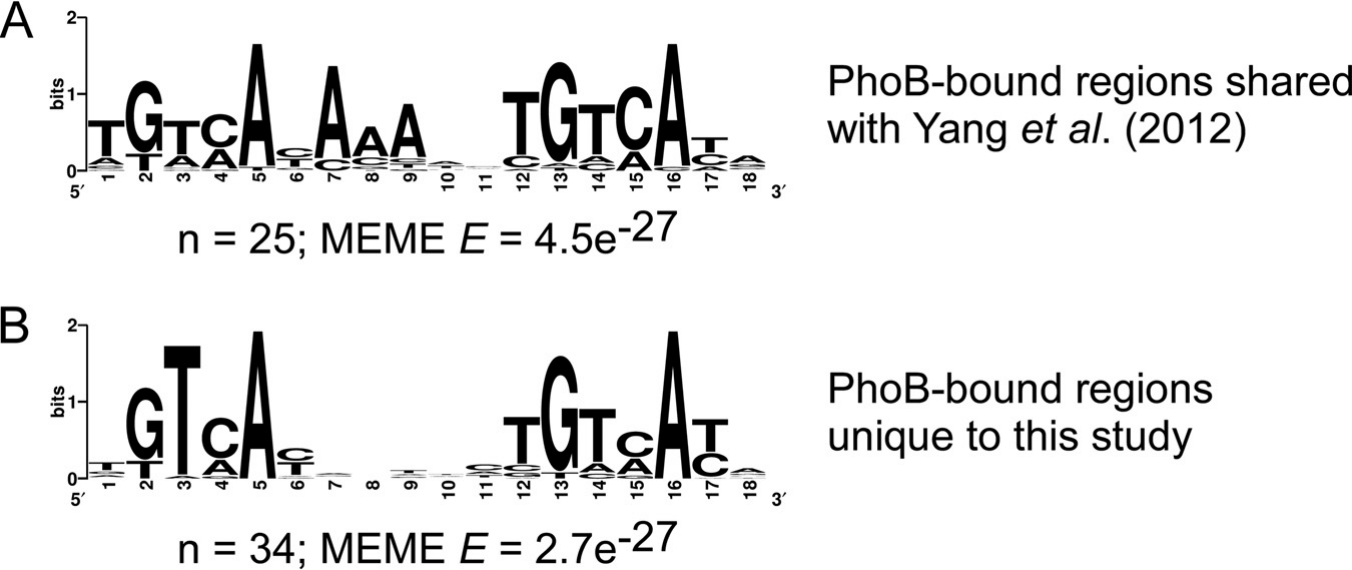
Comparison of ChIP-seq and ChIP-chip data sets. (A) Significantly enriched DNA sequence motif derived from 100-bp regions surrounding each ChIP-seq peak for regions shared between the ChIP-seq data set and a published ChIP-chip data set (43). The number of sites contributing to the motif and the E value determined by MEME are indicated. (B) Significantly enriched DNA sequence motif derived from 100-bp regions surrounding each ChIP-seq peak for regions unique to the ChIP-seq data set, i.e., not found in the published ChIP-chip data set (43). The number of sites contributing to the motif and the E value determined by MEME are indicated.

Our PhoB ChIP-seq data show only modest agreement with an earlier study that identified many putative PhoB binding sites using chromatin immunoprecipitation with microarray technology (ChIP-chip) (43), although both studies are consistent in the lack of signal upstream of *waaH*, *ytfK*, or *psiE*. Of the 43 ChIP-chip peaks identified by Yang et al. (43), 24 are <400 bp from a ChIP-seq peak in our data (Table 1, coordinates in bold), while the remaining 19 ChIP-chip peaks are >2,800 bp from the closest ChIP-seq peak. Even for the 24 ChIP-chip peaks close to ChIP-seq peaks identified in the current study, the peak centers calculated from the two data sets are up to 383 bp apart, and only 13 regions share a motif call between studies (Table 1, motifs in bold). These discrepancies between data sets are probably due, at least in part, to the low resolution of the ChIP-chip data and differences in peak-calling and motif-calling algorithms. It is challenging to determine whether peak calls from each of the two studies represent the same biological binding events. We performed *de novo* motif identification for three sets of peaks: (i) shared, (ii) unique to the current study, and (iii) unique to the study of Yang et al. (43). For shared peaks (i) and peaks unique to the current study (ii), 100 bp of sequence surrounding each ChIP-seq peak center was extracted and analyzed by MEME. In both cases, highly enriched sequence motifs that are close matches to the expected PhoB motif were found (Fig. 3A and B). For sites unique to the Yang et al. data set (iii), the same analysis was performed using both 100-bp and 500-bp windows surrounding the published peak center locations. The resulting motifs were poorly enriched (MEME E values > 1) and bear no similarity to the expected PhoB motif. We conclude that most or all of the 40 regions unique to the current study represent genuine PhoB binding sites, while those unique to the Yang et al. study largely do not.

### Genome-wide binding of PhoB under high-phosphate conditions.

To determine whether PhoB binds any target DNA sites when PhoR is inactive, we repeated the ChIP-seq experiment but grew cells under conditions with high phosphate. We detected only a single PhoB-bound region, the intergenic region upstream of *pstS* (Fig. 2A). This is a well-established site of PhoB binding and was the most enriched PhoB-bound region in the low-phosphate ChIP-seq experiment. As expected, PhoB binding upstream of *pstS* was substantially lower under conditions of high phosphate than under conditions of low phosphate (Fig. 2A). Thus, our data suggest that under conditions of high phosphate, PhoB weakly regulates *pstS* but does not regulate any of its other target genes.

### Reassessing the *pho* regulon.

**FIG 4:**
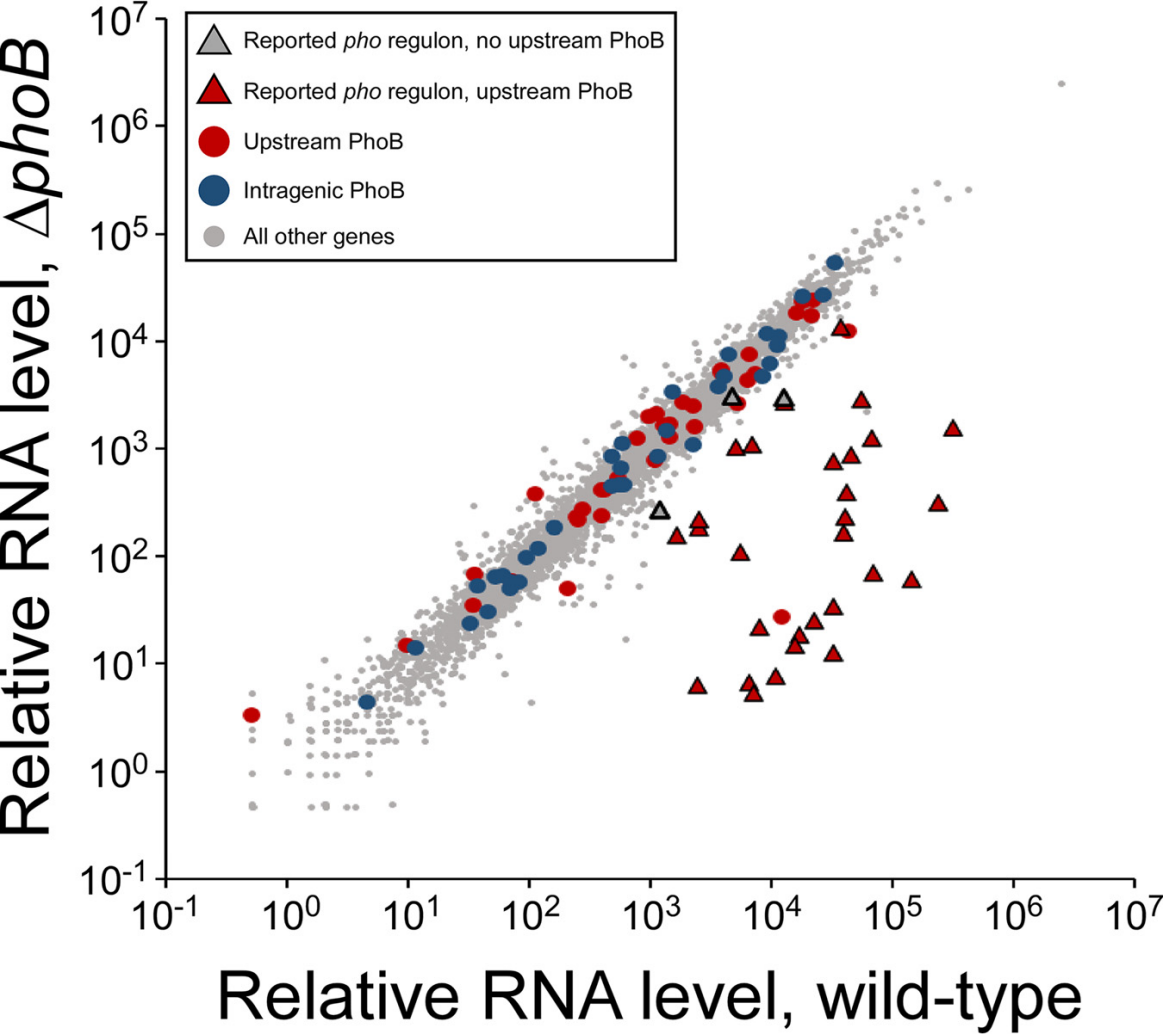
RNA-seq analysis of E. coli wild-type and Δ*phoB* strains. Scatter plot showing relative RNA levels for all genes in wild-type (MG1655/pBAD24) or Δ*phoB* (CDS091/pBAD24) cells. Each data point corresponds to a gene. Triangular data points represent genes previously reported to be in the *pho* regulon, with red fill indicating that the transcript has an upstream PhoB site identified by ChIP-seq and gray fill indicating no upstream site. Red circle data points represent genes not previously reported to be in the *pho* regulon but with upstream PhoB sites identified by ChIP-seq. Blue circle data points represent genes with internal PhoB sites identified by ChIP-seq. All other genes are represented by gray circle data points.

To address whether the detected PhoB sites contribute to transcription regulation, RNA-seq was performed using wild-type and Δ*phoB* strains grown in low-phosphate medium. In total, 181 genes were differentially expressed between the wild-type and Δ*phoB* strains (*P* value ≤ 0.01, >2-fold difference in RNA levels) (Fig. 4; see Table S1 in the supplemental material). We observed significant positive regulation of all 7 reported *pho* regulon operons for which we observed upstream PhoB binding by ChIP-seq, i.e., *phnCDEFGHIJKLMNOP*, *phoH*, *ugpBAECQ*, *pstSCAB*-*phoU*, *phoA*-*psiF*, *phoE*, and *phoBR* (Table 1; Table S1) (positive regulation was observed for all genes in all operons, except for *phoB*, which could not be assessed in the Δ*phoB* strain). We also observed significant positive regulation of *amn* and *yoaI*; ChIP-seq identified PhoB binding upstream of these genes, and although they have not generally been considered part of the *pho* regulon, they have been previously reported as being direct PhoB targets (41, 42).

10.1128/mbio.02535-22.2 TABLE S1 RNA-seq analysis. Download Table S1, XLSX file, 0.2 MB. Copyright © 2023 Fitzgerald et al. 2023 Fitzgerald et al. https://creativecommons.org/licenses/by/4.0/ This content is distributed under the terms of the Creative Commons Attribution 4.0 International license.

We observed significant positive regulation of *ytfK* and *waaH*, reported *pho* regulon genes that lack associated PhoB binding. We conclude that *ytfK* and *waaH* are regulated indirectly by PhoB. In contrast, we did not observe significant regulation of known and predicted *pho* regulon genes *psiE*, *asr*, *eda*, *argP*, and *pitB*; none of these genes had detectable upstream PhoB binding by ChIP-seq. We conclude that *psiE*, *asr*, *eda*, *argP*, and *pitB* are unlikely to be regulatory targets of PhoB.

**FIG 5:**
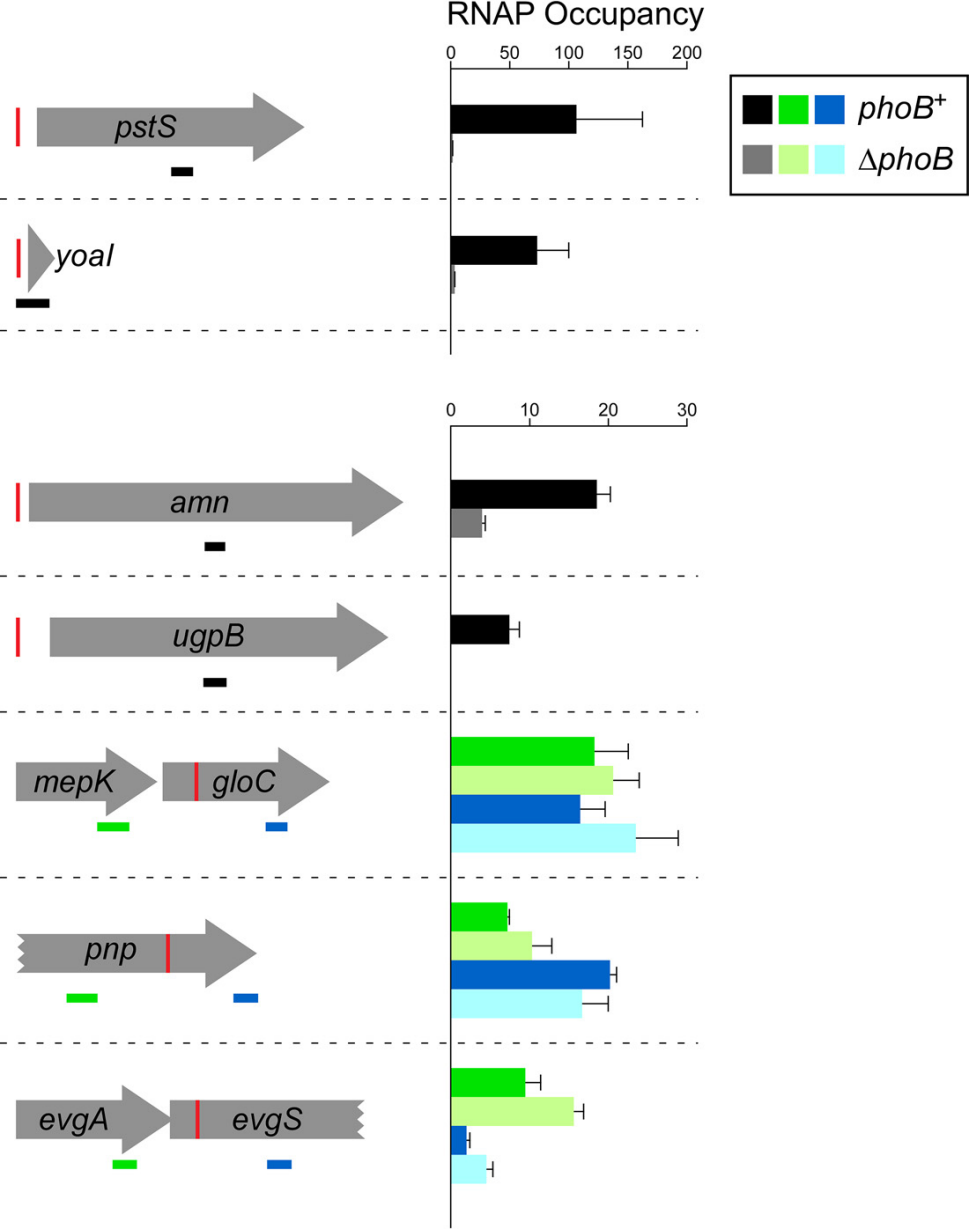
Differences in RNAP (β subunit) occupancy in genes that are potential members of the *pho* regulon. RNAP (β subunit) occupancy measured by ChIP-qPCR (see Materials and Methods for details of how occupancy units are calculated) in wild-type MG1655 (dark-colored bars) and MG1655 Δ*phoB* (CDS091; light-colored bars) for regions within genes that are potential members of the *pho* regulon. Schematics on the left show genes with upstream or internal PhoB sites (red vertical lines). The horizontal bars indicate the positions of PCR amplicons used in ChIP-qPCR, with black bars indicating amplicons within genes that have upstream PhoB sites, green bars indicating amplicons upstream of intragenic PhoB sites, and blue bars indicating amplicons downstream of intragenic PhoB sites. Values are the average of three independent biological replicates; error bars represent 1 standard deviation.

To identify novel *pho* regulon genes, we determined whether any additional genes with associated PhoB binding were significantly differentially expressed between wild-type and Δ*phoB* cells. We observed a significant, >2-fold positive regulation of *cusC* and *yibT* and a significant, >2-fold negative regulation of *feaR* and *agp*, genes with upstream PhoB binding as determined by ChIP-seq. We also observed significant regulation of 6 genes with internal PhoB sites: *yahA*, *gloC*, *pnp*, *evgS*, *eptB*, and *malF*. Although only two of these genes (*yahA* and *evgS*) were differentially expressed >2-fold between wild-type and Δ*phoB* cells, all but *yahA* were more highly expressed in Δ*phoB* cells than wild-type cells. We hypothesized that PhoB represses transcription of these genes by acting as a roadblock for RNAP. To test this hypothesis, we grew wild-type and Δ*phoB* cells under phosphate-limiting conditions and measured RNAP (β subunit) occupancy upstream and downstream of the PhoB sites within the *gloC*, *pnp*, and *evgS* genes by using ChIP-quantitative PCR (ChIP-qPCR). For controls, we measured RNAP occupancy within the *pstS* and *ugpB* genes, confirmed members of the *pho* regulon. We also measured RNAP occupancy within the *yoaI* and *amn* genes that the combined RNA-seq and ChIP-seq data suggested are members of the *pho* regulon. As expected, we observed substantially higher RNAP occupancy within *pstS* and *ugpB* in wild-type cells than in Δ*phoB* cells (Fig. 5). Moreover, we observed substantially higher RNAP occupancy within *yoaI* and *amn* in wild-type cells than in Δ*phoB* cells (Fig. 5), supporting the idea that these genes are part of the *pho* regulon. For the three genes with intragenic PhoB sites, we reasoned that if PhoB acts as a roadblock to the elongation of RNAP, RNAP occupancy downstream of PhoB sites would increase in Δ*phoB* cells relative to that in wild-type cells and relative to any change in RNAP occupancy upstream of the PhoB site. However, we did not observe significant increases in relative RNAP occupancy downstream of PhoB sites for any of the three genes (Fig. 5) (*P > *0.2; see Materials and Methods for details of the statistical analysis), suggesting that PhoB regulation of these genes is indirect.

### PhoB-dependent recruitment of initiating RNAP.

The majority of the PhoB binding sites identified by ChIP-seq were not associated with regulation detectable by RNA-seq. We hypothesized that this could be due to three reasons: (i) the binding sites are nonregulatory, (ii) regulation is condition specific and/or requires additional factors, or (iii) PhoB regulates transcription of short, unstable, noncoding RNAs that are not detectable by conventional RNA-seq. To test the latter possibility, we used ChIP-seq to measure the association of σ^70^ in regions close to PhoB binding sites. σ^70^ is rapidly released from RNAP upon the transition from transcription initiation to elongation (44); thus, σ^70^ occupancy on DNA, as measured by ChIP-seq, is an indication of the level of association of initiating RNAP with DNA. Since transcription initiation occurs prior to RNA processing, σ^70^ occupancy can be observed even at promoters of highly unstable RNAs (45).

**FIG 6:**
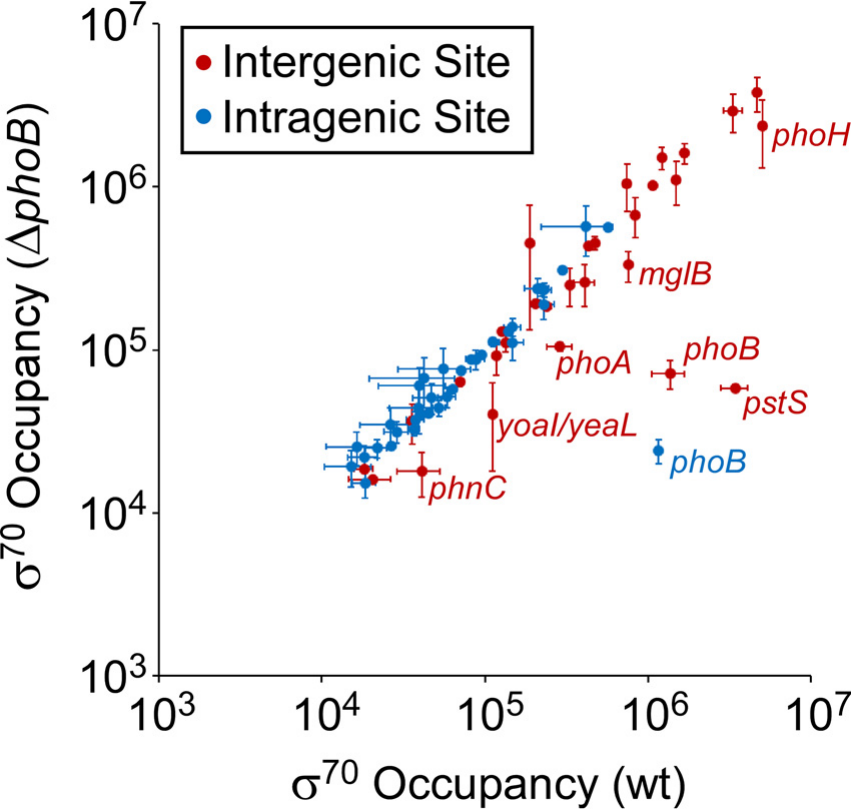
Differences in σ^70^ occupancy around PhoB binding sites between wild-type and Δ*phoB* cells. The scatter plot shows normalized σ^70^ occupancy in wild-type MG1655 and MG1655 Δ*phoB* (DMF84) for the 400-bp regions surrounding PhoB binding sites identified by ChIP-seq. Each data point represents a PhoB binding site. Intergenic binding sites are indicated by red data points, and intragenic binding sites are indicated by blue data points. Genes associated with PhoB binding sites are labeled with the gene name in cases where σ^70^ occupancy differs >2-fold between wild-type and Δ*phoB* cells. Values are the average of two independent biological replicates; error bars represent ±1 standard deviation.

To measure the effects of PhoB on RNAP holoenzyme recruitment, we performed ChIP-seq of σ^70^ in wild-type and Δ*phoB* strains grown in low-phosphate medium. Normalized σ^70^ occupancy was calculated for 400-bp windows surrounding each PhoB binding site to systematically assess σ^70^ binding at these sites (Fig. 6). Three PhoB binding sites showed large reductions (>19-fold) in σ^70^ occupancy in the Δ*phoB* strain relative to that in the wild type. Two of these sites are associated with the *phoB* gene itself; σ^70^ occupancy measurements at these sites are impacted by the loss of associated DNA sequence resulting from deletion of *phoB*. The third PhoB site is the regulatory site upstream of *pstS*. We conclude that PhoB activates *pstS* transcription at the level of RNAP recruitment, as suggested by structural models of the DNA-PhoB-RNAP complex (46–48). PhoB binding sites upstream of *yoaI*, *phoA*, *mglB*, *phnC*, and *phoH* showed >2-fold lower σ^70^ occupancy in the Δ*phoB* strain than in the wild type, suggesting that PhoB recruits initiating RNAP to these promoters. These data are largely consistent with the RNA-seq data showing >2-fold differential expression of *yoaI*, *phoA*, *phnC*, and *yoaI* between wild-type and Δ*phoB* cells (Table 1). For most other PhoB sites, including almost all intragenic sites, σ^70^ occupancy was low in both wild-type and Δ*phoB* strains (Fig. 6), strongly suggesting that these sites are not associated with active promoters under the growth conditions used. The remaining sites were associated with substantial σ^70^ occupancy, which was similar in both wild-type and Δ*phoB* strains, suggesting that they are close to active promoters whose activity is independent of PhoB under the conditions tested.

### H-NS coassociates with many intragenic PhoB sites but does not block RNAP recruitment.

**FIG 7:**
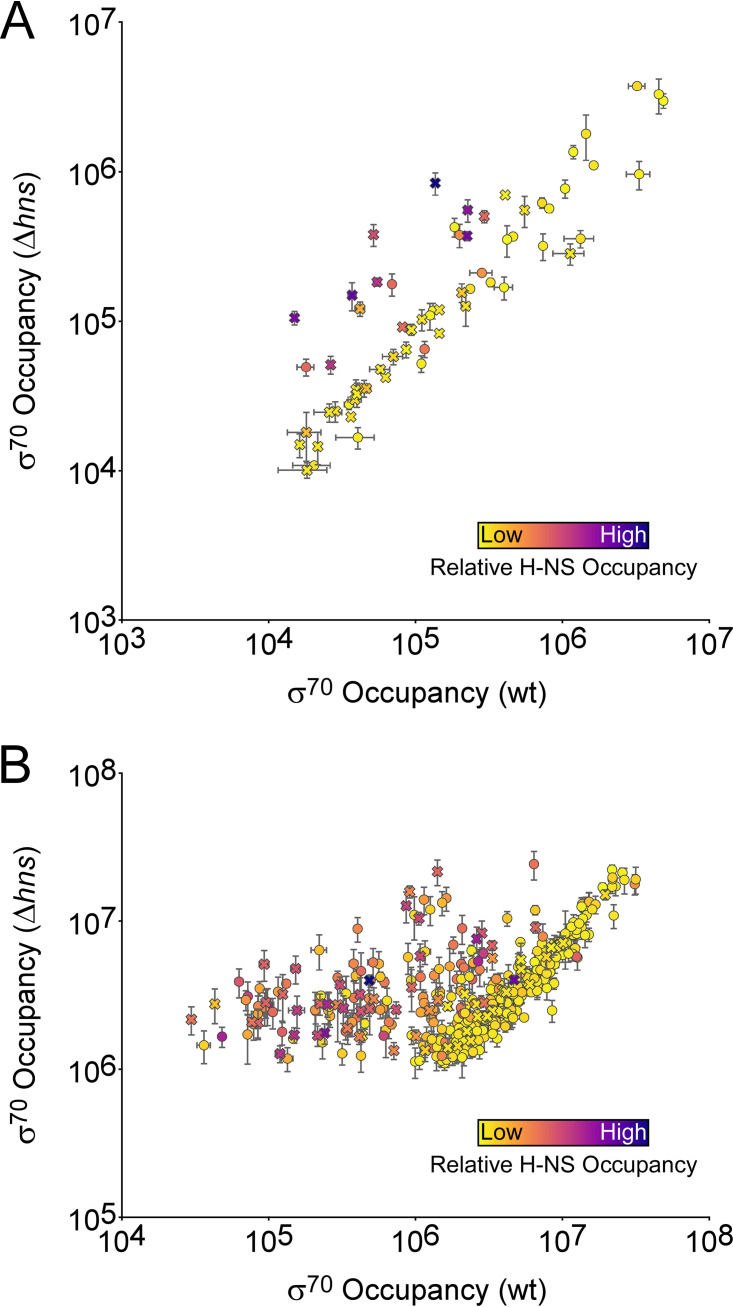
H-NS suppresses transcription from many promoters. (A) Scatter plot showing normalized σ^70^ occupancy in wild-type MG1655 and MG1655 Δ*hns* (AMD565a) for the 400-bp regions surrounding PhoB binding sites identified by ChIP-seq. Each data point represents a PhoB binding site. The color of each data point indicates the level of H-NS occupancy at the corresponding site (49). Intragenic PhoB sites are represented by crosses; intergenic PhoB sites are represented by circles. (B) Scatter plot showing normalized σ^70^ occupancy in wild-type MG1655 and MG1655 Δ*hns* (AMD565a) for all σ^70^ binding sites identified by ChIP-seq from MG1655 Δ*hns* (AMD565a) cells. The color of each data point indicates the level of H-NS occupancy at the corresponding site (49). Intragenic PhoB sites are represented by crosses; intergenic PhoB sites are represented by circles. For both panels A and B, values are the average of two independent biological replicates; error bars represent ±1 standard deviation.

**FIG 8:**
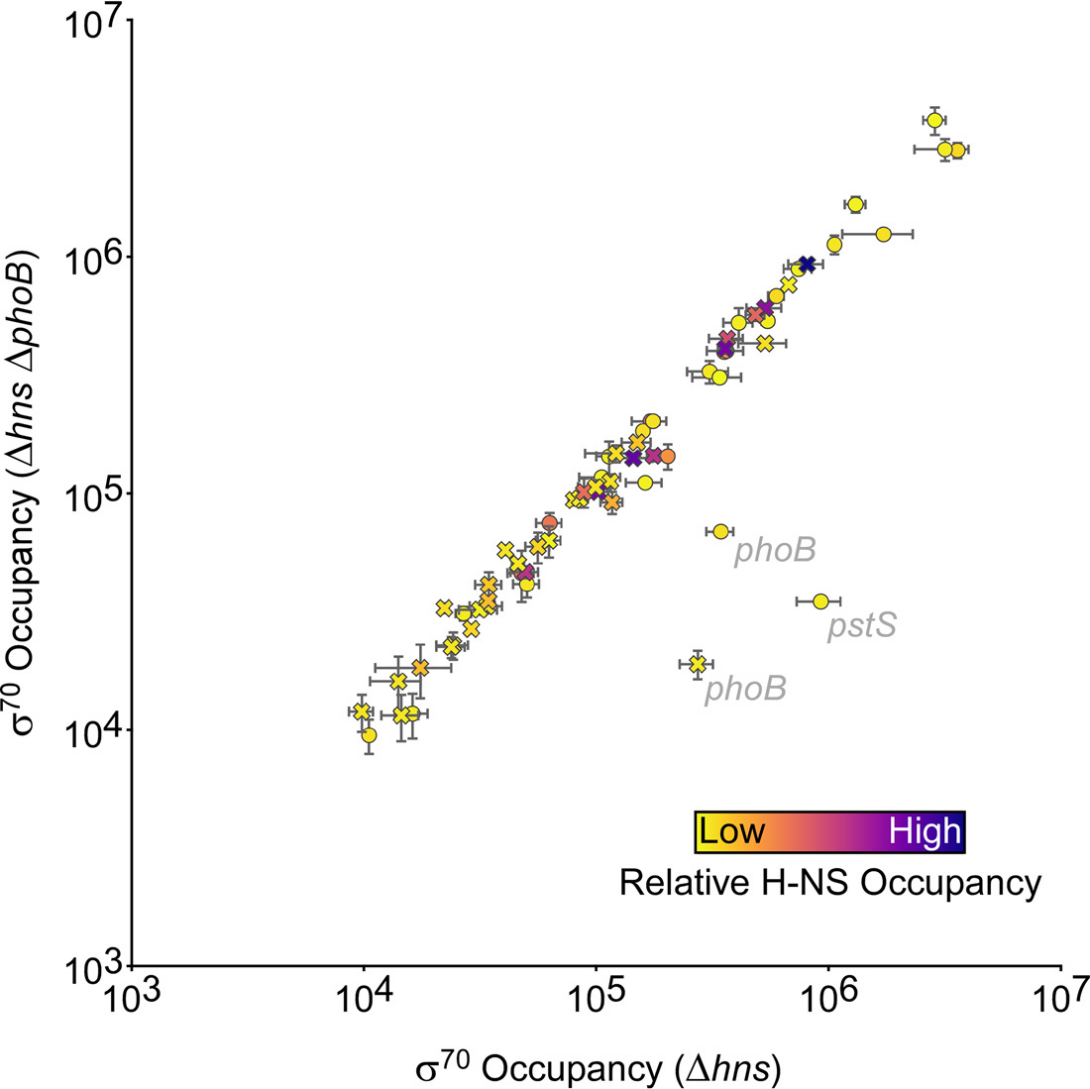
H-NS does not suppress PhoB-dependent effects on recruitment of initiating RNA polymerase. The scatter plot shows normalized σ^70^ occupancy in wild-type MG1655 Δ*hns* (AMD565a) and MG1655 Δ*hns* Δ*phoB* (DMF85) for the 400-bp regions surrounding PhoB binding sites identified by ChIP-seq. Each data point represents a PhoB binding site. The color of each data point indicates the level of H-NS occupancy at the corresponding site (49). Intragenic PhoB sites are represented by crosses; intergenic PhoB sites are represented by circles. Genes associated with PhoB binding sites are indicated in cases where σ^70^ occupancy differs >2-fold between MG1655 Δ*hns* (AMD565a) and MG1655 Δ*hns* Δ*phoB* (DMF85) cells. Values are the average of two independent biological replicates; error bars represent ±1 standard deviation.

We noted that 18 PhoB sites (12 intragenic and 6 intergenic), representing 28% of all sites identified by ChIP-seq, are in regions bound by the nucleoid-associated protein H-NS (49). Thus, PhoB sites are significantly enriched in H-NS-bound regions, which represent only 17% of the genome (binomial test, *P = *0.02). Since H-NS is known to silence transcription (50), we hypothesized that the lack of detectable PhoB-dependent regulation at some sites may be due to the silencing effects of H-NS. To test this hypothesis, we repeated the σ^70^ ChIP-seq experiment in Δ*hns* and Δ*hns* Δ*phoB* strains. Comparison of σ^70^ occupancy between wild-type and Δ*hns* strains revealed substantially increased occupancy around some PhoB binding sites in the Δ*hns* strain, with most of these sites being intragenic (Fig. 7A). Indeed, we observed widespread increases in σ^70^ occupancy at promoters genome-wide in the Δ*hns* strain relative to that in the wild type; most of the promoters showing increased σ^70^ association are located in regions of high H-NS occupancy (Fig. 7B) (49). These data are consistent with our earlier study showing widespread transcriptional silencing by H-NS, particularly within genes (45). We next compared σ^70^ occupancies around PhoB binding sites between Δ*hns* and Δ*hns* Δ*phoB* strains (Fig. 8). As for *hns*^+^ cells, the only large differences (>5-fold) in σ^70^ occupancy were associated with the PhoB sites at *phoB* and *pstS*. Interestingly, we did not observe differences of >1.5-fold in σ^70^ occupancy at any other sites, including the sites upstream of *yoaI*, *phoA*, *mglB*, *phnC*, and *phoH*. However, the differences in σ^70^ occupancy that we observed for *phoB* and *pstS* sites were between 2- and 4-fold lower than the differences observed in the *hns*^+^ strains at the same sites. Hence, the more subtle differences in σ^70^ occupancy observed at the sites upstream of *yoaI*, *phoA*, *phnC*, and *phoH* in the *hns*^+^ strains may have escaped detection in the Δ*hns* strains. The lower effect of PhoB on σ^70^ occupancy in the Δ*hns* strain background may be due to the large-scale redeployment of RNAP that occurs in the absence of H-NS (51).

While PhoB sites are enriched within H-NS-bound regions, H-NS does not appear to modulate PhoB activity at any site. We hypothesized that the enrichment of PhoB binding within H-NS-bound regions is due simply to the nucleotide content of the PhoB binding site; like H-NS-bound regions, the PhoB binding site has a higher A/T content than the genome as a whole. To test this hypothesis, we scrambled the sequence of every PhoB binding site identified by ChIP-seq. We then derived a position weight matrix (PWM) from these scrambled sites and scored every genomic sequence for a match to this PWM. Strikingly, 36% of the top 1,000 scoring positions are within regions bound by H-NS (49). We conclude that the enrichment of PhoB binding sites within H-NS-bound regions is likely due to the A/T-rich nature of the binding motif.

### Sequence conservation of PhoB binding sites.

**FIG 9:**
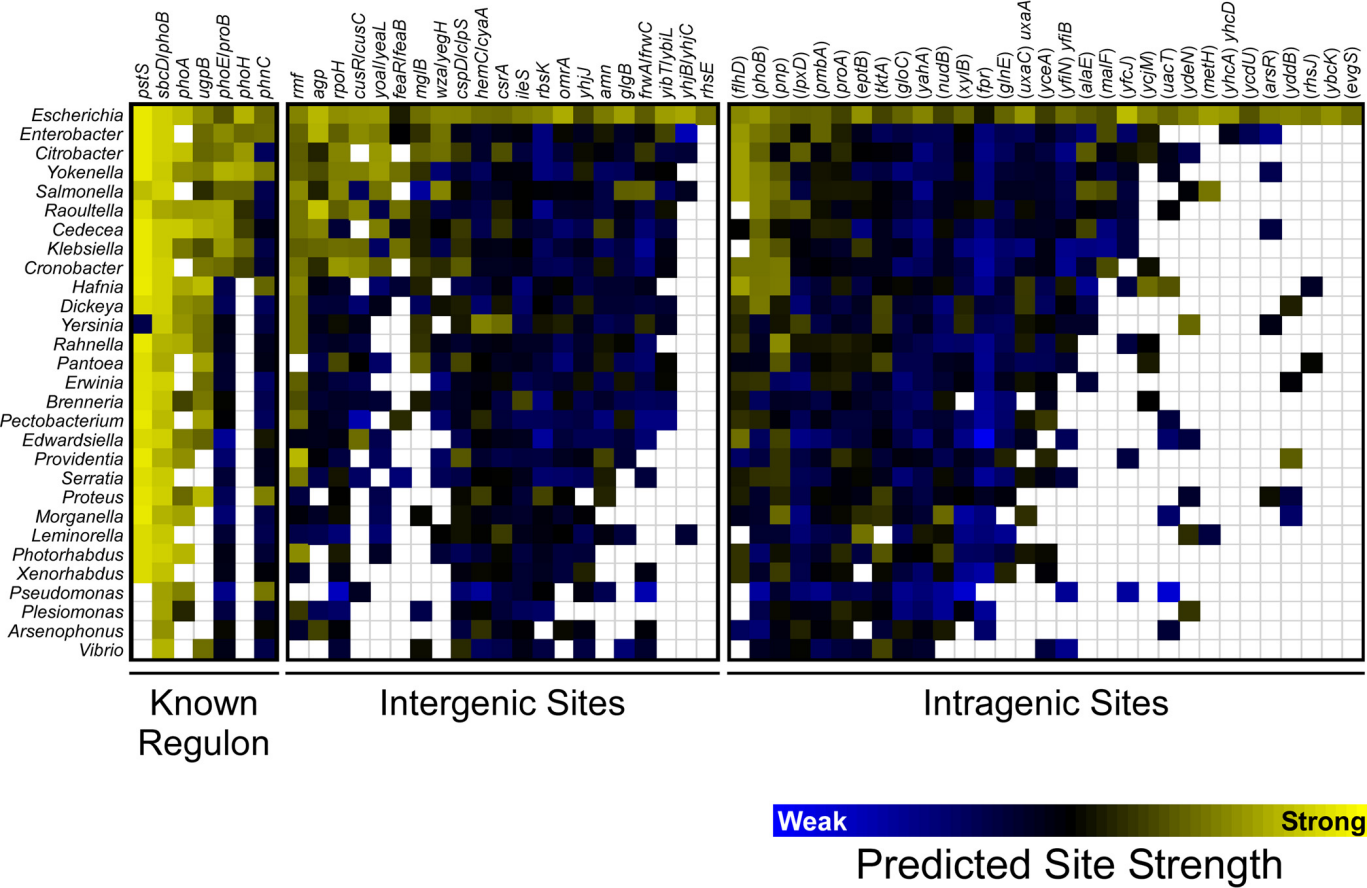
Conservation of PhoB binding sites across gammaproteobacterial species. A heat map shows conservation of PhoB binding sites across selected gammaproteobacterial species. Columns represent PhoB binding sites from E. coli, divided into known *pho* regulon binding sites, intergenic sites, and intragenic sites. The associated genes are indicated above each column. Rows represent species for different gammaproteobacterial genera, as indicated to the left of each row. The color of each square indicates the predicted strength of the best-scoring putative PhoB binding site in a region that is homologous to the corresponding region in E. coli. Binding site strength was predicted using a position weight matrix derived from the E. coli PhoB binding site motif (Fig. 2B). The color scale is shown below the heat map, with yellow indicating stronger predicted binding site strength and blue indicating weaker predicted binding site strength. White indicates the absence of a homologous region in the indicated species.

Sequence conservation of a DNA binding site is often an indication that the site is functional (52). We determined the sequence conservation of the 59 E. coli PhoB sites identified by ChIP-seq for which we could identify an instance of the PhoB binding motif. Specifically, we scored homologous regions from 29 diverse gammaproteobacterial species for matches to the PhoB binding site motif (Fig. 2B). The DNA-binding domain of PhoB is highly conserved across these species (Fig. S1). As shown in Fig. 9, the PhoB binding sites upstream of *pstS*, *phoB*, *phoA*, and *ugpB* are broadly conserved. The PhoB binding sites upstream of *phoE* and *phoH* are conserved, albeit to a lesser degree. The PhoB binding site upstream of *phnC* is conserved in only a few species, suggesting that *phnC* is not a core member of the *pho* regulon. Among the novel PhoB binding sites, the best conserved is the site upstream of *rmf*, with strong matches to the PhoB DNA binding motif found upstream of *rmf* in most species analyzed. We detected PhoB upstream of *rmf* by ChIP-seq (Table 1) but did not detect significant PhoB-dependent regulation at the level of RNA abundance or RNAP recruitment. PhoB binding sites upstream of *agp*, *rpoH*, *cusR*/*cusC*, and *yoaI* were also conserved, albeit to a lesser degree, similar to sites upstream of *phoE* and *phoH*. The remaining intergenic PhoB sites were not well conserved, with most having few or no strong matches to the PhoB DNA binding motif outside of E. coli. Lastly, we examined conservation of intragenic PhoB sites. In most cases, these sites had little or no conservation; however, PhoB sites within *flhD*, *phoB*, and *pnp* were conserved among roughly half the species examined. This conservation may reflect a conserved function for the PhoB binding site or could be due to sequence constraints on the codons.

10.1128/mbio.02535-22.1 FIG S1 Alignment of PhoB protein sequences used for phylogenetic analysis of binding sites. PhoB protein sequences from 29 gammaproteobacterial species. Amino acids are color coded by chemical property; amino acids are colored only if they are identical to the equivalent position in E. coli. Asterisks under the alignment indicate amino acids reported to contact DNA (32). The percent coverage (cov) and identity (id) relative to the E. coli sequence are indicated next to the genus name. Download FIG S1, PDF file, 0.2 MB. Copyright © 2023 Fitzgerald et al. 2023 Fitzgerald et al. https://creativecommons.org/licenses/by/4.0/ This content is distributed under the terms of the Creative Commons Attribution 4.0 International license.

## DISCUSSION

### Comprehensive reassessment of the *pho* regulon in E. coli and beyond.

By combining ChIP-seq and RNA-seq, we were able to reassess the *pho* regulon, with high-resolution assignment of PhoB binding sites. As described above, the sensitivity and resolution of an earlier ChIP-chip study were substantially lower, precluding a comprehensive reassessment of the *pho* regulon (43). Previous studies disagree on which genes constitute the *pho* regulon in E. coli; however, most studies agree that the *pho* regulon includes the following operons: *pstSCAB*-*phoU*, *phoA*, *phoH*, *phnCDEFGHIJKLMNOP*, *phoBR*, *phoE*, *ytfK*, *ugpBAECQ*, and *psiE* (30, 33). *waaH* is considered in some studies to be in the *pho* regulon (33). Our data are largely consistent with these assignments but provide strong evidence against *ytfK*, *psiE*, and *waaH* being members of the *pho* regulon. Specifically, we did not detect PhoB binding near any of these genes (Table 1), and we did not detect PhoB-dependent regulation of *psiE* (see Table S1 in the supplemental material). It is formally possible that the C-terminal FLAG tags on PhoB altered its binding specificity or reduced its affinity for DNA such that we failed to detect binding to sites upstream of *ytfK*, *psiE*, and *waaH*. Nonetheless, in such a scenario, these binding sites would presumably have relatively low affinity for PhoB.

Our data also rule out several other putative *pho* regulon genes, namely, *asr*, *eda*, *argP*, and *pitB*, which were not associated with detectable PhoB binding or PhoB-dependent regulation (40, 53–57). Similarly, we did not detect binding of PhoB upstream of the small RNA (sRNA)-encoding gene *esrL*, despite a recent report of PhoB binding to this region in enteropathogenic E. coli, with the sequence of the reported PhoB site being identical in E. coli K-12 (58). In contrast, our ChIP-seq data support the assignment of *amn* and *yoaI* as *pho* regulon members, as has previously been suggested based on limited experimental evidence (40–42). Our ChIP-seq and RNA-seq data identify novel *pho* regulon members with confidence, namely, *cusC*, *feaR*, *yibT*, and *agp*. These genes all have PhoB binding sites upstream and were significantly differentially expressed >2-fold between wild-type and Δ*phoB* strains. We note that direct positive regulation of *cusC* and direct negative regulation of *feaR* by PhoB have been suggested previously (43). Our data provide no evidence to suggest that there are unannotated transcripts regulated by PhoB or transcripts whose regulation by PhoB is masked by H-NS (Fig. 6 and 8). Lastly, our data do not support direct regulation by PhoB sites located within genes (Fig. 5).

Phylogenetic analysis of PhoB binding sites highlights a highly conserved set of *pho* regulon genes within the gammaproteobacteria, namely, *pstS*, *phoB*, *phoA*, *ugpB*, and associated operonic genes (Fig. 9). Consistent with this, direct PhoB regulation of the *pstS* and *phoB* transcripts has been described for the more distantly related alphaproteobacterium Caulobacter crescentus (59). *phoE*, *phoH*, and *yoaI* represent a second set of conserved *pho* regulon genes, although their conservation is more phylogenetically restricted. Interestingly, while PhoB regulation of *phnC* and associated operonic genes does not appear to be widely conserved, we did observe evidence for strong PhoB sites upstream of *phnC* in a small set of species, and *phnC* is known to be a direct regulatory target of PhoB in C. crescentus (59), suggesting that *phnC* may have a niche-specific function in phosphate homeostasis.

The phylogenetic pattern of PhoB binding site conservation for sites upstream of *rmf*, *agp*, *rpoH*, and *cusR*/*cusC* suggests that these genes may be part of the conserved *pho* regulon. We observed significant differential expression of *cusC* and *agp* between wild-type and Δ*phoB* cells. In contrast, we did not observe significant differential expression of *rmf* or *rpoH*. We speculate that regulation of *rmf* and *rpoH* by PhoB is integrated with regulation by other TFs, such that PhoB-dependent changes in expression are detectable only under specific growth conditions. Consistent with this idea, transcription of *rmf* has been shown to be regulated by ppGpp (60, 61) and cyclic AMP receptor protein (CRP) (62) and possibly by additional TFs (63) and diverse stress conditions (64). PhoB binding sites upstream of *agp*, *rpoH*, and *cusR*/*cusC* are conserved in largely the same set of species as binding sites upstream of *phoE*, *phoH*, and *yoaI*, suggesting that these species share a set of *pho* regulon genes.

### Most intragenic PhoB sites appear to be nonfunctional and are not under selective pressure.

Our data argue against intragenic PhoB sites having regulatory activities of the types that have been described previously for intragenic TF sites, specifically, regulation of transcription from an intragenic promoter (5, 7–12) or regulation of the overlapping gene either by roadblock repression or a novel mechanism (3, 15–18). Indeed, most intragenic PhoB sites are associated with little or no local σ^70^ binding (Fig. 6), indicating that PhoB binding alone is insufficient to recruit RNAP. Thus, it is likely that RNAP:σ^70^-interacting promoter elements are also necessary for PhoB-dependent recruitment of RNAP. Moreover, the spacing between PhoB sites and core promoter elements is likely to be important in determining whether PhoB recruits RNAP, since even intragenic PhoB sites that are close to intragenic promoters (i.e., those associated with high ChIP-seq signal for σ^70^) show no difference in σ^70^ occupancy upon deletion of *phoB*. Consistent with this idea, structural models of the PhoB-RNAP-DNA complex formed at PhoB-activated promoters support strict spacing requirements between the *pho* box and core promoter elements (46–48, 65).

Widespread intragenic TF binding is emerging as a common phenomenon as more TFs are mapped using ChIP-seq (2–6, 66). Similarly, many σ factors have been shown to bind and initiate transcription from large numbers of intragenic promoters (8, 19, 20, 67–70). In the majority of cases tested, these intragenic binding sites are poorly conserved (69, 71), as is the case for intragenic PhoB sites (Fig. 9). Based on the lack of detectable transcriptional activity, and the limited conservation of intragenic PhoB sites, we speculate that intragenic TF sites often arise due to genetic drift or selective pressures on overlapping sequences such as codons. Consistent with this idea, intragenic PhoB sites tend to be weaker (lower ChIP-seq enrichment) than intergenic sites (Mann-Whitney U test, *P = *0.005). A previous study showed that the predicted number of intragenic binding sites for many bacterial TFs is the same in actual genome sequences as it is in randomized genome sequences, suggesting that intragenic TF binding sites are common and arise largely due to genetic drift (28). We further speculate that the fitness cost of intragenic PhoB sites is low. Intragenic TF binding sites can therefore be considered genomic “noise.” Interestingly, the vast majority of PhoB binding events detected in C. crescentus are intergenic (59), suggesting that intragenic PhoB binding in C. crescentus may be associated with a fitness cost. Finally, we cannot rule out the possibility that intragenic PhoB sites in E. coli are functional. For example, they could contribute, *en masse*, to titration of PhoB, they could facilitate DNA looping that impacts chromosome structure, as has been suggested for some TFs (22–27), or they could regulate transcription by unknown mechanisms.

We have comprehensively mapped the PhoB regulon by assessing PhoB binding, PhoB-dependent transcriptome changes, and PhoB-dependent RNAP recruitment. We identified novel *pho* regulon members, some of which are modestly conserved across other genera, and identified many seemingly nonfunctional PhoB binding sites inside genes. We conclude that a combination of binding site information (e.g., ChIP-seq) and regulatory information (e.g., RNA-seq) is required to accurately define the regulons of most TFs.

## MATERIALS AND METHODS

### Strains and plasmids.

E. coli MG1655 and its derivatives were used for this study. The strains and plasmid used are listed in Table 2. All oligonucleotides used are listed in Table S2 in the supplemental material. For ChIP-seq, PhoB was C-terminally epitope tagged with a 3×FLAG tag. The tag was inserted at the native *phoB* locus by use of FRUIT recombineering (72) using oligonucleotides JW2973 and JW2974. MG1655 Δ*phoB* (DMF84) and Δ*hns* (AMD565a) strains were constructed by P1 transduction from Keio collection strains (73) into MG1655. The Kan^r^ genes were removed by FLP-recombinase expressed from pCP20, as previously described (74). The MG1655 Δ*hns* Δ*phoB* strain was made in the same manner, with deletions introduced sequentially. MG1655 Δ*phoB* (CDS091) was constructed by use of FRUIT (72) using oligonucleotides JW6280, JW6281, JW6294, and JW6295. Note that there are two MG1655 Δ*phoB* strains used in this study; DMF34 was used for σ^70^ ChIP-seq experiments, and CDS091 was used for qRT-PCR, RNA-seq, and RNAP ChIP-qPCR experiments.

**TABLE 2:** List of strains and plasmids used in this study

Strain or plasmid	Description	Source or reference
Strains		
MG1655	Escherichia coli MG1655	89
DMF34	MG1655 *phoB*-FLAG_3_	This study
DMF84	MG1655 Δ*phoB*	This study
AMD565a	MG1655 Δ*hns*	This study
DMF85	MG1655 Δ*hns* Δ*phoB*	This study
CDS091	MG1655 Δ*phoB*	This study
Plasmid		
pBAD24	Empty vector for arabinose-inducible expression	90
pCP20	Expression of FLP-recombinase	74

10.1128/mbio.02535-22.3 TABLE S2 List of oligonucleotides used in this study. Download Table S2, XLSX file, 0.01 MB. Copyright © 2023 Fitzgerald et al. 2023 Fitzgerald et al. https://creativecommons.org/licenses/by/4.0/ This content is distributed under the terms of the Creative Commons Attribution 4.0 International license.

### qRT-PCR.

qRT-PCR was performed based on a previous study (69). MG1655/pBAD24, CDS091 (MG1655 Δ*phoB*)/pBAD24, or DMF34/pBAD24 cells were grown at 37°C with aeration in MOPS (morpholinepropanesulfonic acid) minimal medium with 0.2 mM K_2_PO_4_, 0.4% glucose, and 100 μg/mL ampicillin to an optical density at 600 nm (OD_600_) of 0.5 to 0.6. Arabinose was added to a final concentration of 0.2% for 7 min or 20 min, with one or two of the three replicate samples for each strain receiving arabinose for 7 min. Thus, the replicate samples were not always consistent with respect to the extent of growth after arabinose addition. However, since arabinose is not expected to affect *pstS* expression, all samples for a single strain were treated as replicates regardless of whether the cells were grown for 7 min or 20 min after arabinose addition. RNA was prepared as described for RNA-seq. RNA was reverse transcribed using SuperScript III reverse transcriptase (Invitrogen) according to the manufacturer’s instructions. A control reaction omitting reverse transcriptase was performed. One percent of the cDNA (or negative control) was used as a template in a quantitative real-time PCR using an Applied Biosystems 7500 fast real-time PCR machine, with primers JW156 and JW157 for amplifying the *minD* control gene and primers JW7802 and JW7803 for amplifying *pstS*. Relative expression of *pstS* was determined by the Δ*C_T_* method, with normalization to *minD* expression.

### ChIP-seq.

For low-phosphate growth experiments, cells were grown at 37°C with aeration in MOPS minimal medium with 0.2 mM K_2_HPO_4_ and 0.4% glucose, as previously described (37, 75). For high-phosphate growth experiments, cells were grown in MOPS minimal medium with 1.32 mM K_2_HPO_4_ and 0.4% glucose. Subcultures were inoculated 1:100 and grown at 37°C with aeration to an OD_600_ of 0.5 to 0.7. ChIP-seq libraries were prepared as previously described (76). Libraries were prepared from two biological replicate cultures for each experimental group. For DMF34 (MG1655 *phoB*-FLAG_3_) or MG1655, PhoB-FLAG_3_ (or the untagged control) was immunoprecipitated with 2 μL of anti-FLAG M2 monoclonal antibody (Sigma). For MG1655, DMF84 (Δ*phoB*), AMD565a (MG1655 Δ*hns*), and DMF85 (MG1655 Δ*phoB* Δ*hns*), σ^70^ was immunoprecipitated with 1 μL of anti-σ^70^ antibody (Neoclone). Libraries were sequenced on a HiSeq 2000 (Illumina) by the University at Buffalo Next-Generation Sequencing Core Facility or on a NextSeq (Illumina) by the Wadsworth Center Applied Genomic Technologies Core Facility.

### Analysis of PhoB-FLAG_3_ ChIP-seq data.

Duplicate ChIP-seq data for (i) MG1655 *phoB*-FLAG_3_ (DMF34) grown in low-phosphate conditions, (ii) untagged MG1655 grown in low-phosphate conditions, and (iii) MG1655 *phoB*-FLAG_3_ (DMF34) grown in high-phosphate conditions were aligned to the E. coli MG1655 genome (GenBank accession no. NC_000913.3) using CLC Genomics Workbench (version 8). ChIP-seq peaks were called using a previously described analysis pipeline (8).

### Analysis of RNAP occupancy around PhoB binding sites.

Wild-type MG1655 and MG1655 Δ*phoB* (CDS091) cells were grown at 37°C with aeration to an OD_600_ of 0.6 to 0.7 in MOPS minimal medium with 0.2 mM K_2_HPO_4_ and 0.4% glucose, as previously described (37, 75). ChIP was performed as previously described (76) using 1 μL anti-β (RNA polymerase subunit) antibody (BioLegend catalog no. 663903). ChIP and input samples were analyzed using an ABI 7500 fast real-time PCR machine. Enrichment of ChIP samples was calculated relative to a control region within *bglB*, which is transcriptionally silent. RNAP occupancy units represent background-subtracted fold enrichment over the control region. Oligonucleotides used for qPCR were JW125 + JW126 (*bglB*), JW10937 + JW10938 (*yoaI*), JW10939 + JW10940 (*amn*), JW10941 + JW10942 (*pstS*), JW10943 + JW10944 (*ugpB*), JW10945 + JW10946 (*mepK*), JW10947 + JW10948 (*gloC*), JW10949 + JW10950 (*evgA*), JW10951 + JW10952 (*evgS*), JW10953 + JW10954 (*pnp* upstream), and JW10955 + JW10956 (*pnp* downstream). For statistical analysis of relative changes in RNAP occupancy upstream and downstream of intragenic PhoB sites, we used the mean and standard deviation values for ChIP-qPCR occupancy to generate 1,000 simulations for occupancy at each region tested. We then determined how frequently the ratio of predicted RNAP occupancy downstream to predicted RNAP occupancy upstream of an intragenic PhoB site was higher in Δ*phoB* cells than wild-type cells. We repeated this simulation 10 times to estimate a *P* value for each of the three intragenic PhoB sites tested.

### Analysis of σ^70^ occupancy around PhoB binding sites.

Duplicate ChIP-seq data for σ^70^ from wild-type MG1655, MG1655 Δ*phoB* (DMF84), MG1655 Δ*hns* (AMD565a), and MG1655 Δ*hns* Δ*phoB* (DMF85) were aligned to the E. coli MG1655 genome (GenBank accession no. NC_000913.3) using Rockhopper (version 2.03; default parameters) (77), which also calculated the depth of sequence coverage at all genomic positions on each strand, normalized for total sequence read count. A custom Python script was used to determine the relative sequence read coverage from each σ^70^ ChIP-seq data set in 400-bp windows centered on each PhoB ChIP-seq peak (coordinates listed in Table 1).

### Analysis of σ^70^ occupancy at promoters in wild-type and Δ*hns* strains.

Duplicate ChIP-seq data for σ^70^ for wild-type MG1655 or MG1655 Δ*hns* (AMD565a) were aligned to the E. coli MG1655 genome (GenBank accession no. NC_000913.3) using CLC Genomics Workbench (version 8). ChIP-seq peaks were called using a previously described analysis pipeline (8). A custom Python script was used to determine the relative sequence read coverage from each σ^70^ ChIP-seq data set in 50-bp windows centered on each σ^70^ ChIP-seq peak from MG1655 Δ*hns* (AMD565a).

### Determining H-NS occupancy from published ChIP-seq data.

H-NS ChIP-seq occupancy was determined from published data (49). Specifically, genome coordinates for σ^70^ ChIP-seq peaks were converted from NCBI genome sequence version U00096.3 to U00096.2 at https://biocyc.org/ECOLI/map-seq-coords-form?chromosome=COLI-K12. H-NS occupancy was determined as the average from four normalized sequence read coverage files available from EBI ArrayExpress under accession number E-MTAB-332.

### RNA-seq.

MG1655/pBAD24 or CDS091 (MG1655 Δ*phoB*)/pBAD24 cells were grown at 37°C with aeration in MOPS minimal medium with 0.2 mM K_2_PO_4_, 0.4% glucose, and 100 μg/mL ampicillin to an OD_600_ of 0.5 to 0.6. Arabinose was added to a final concentration of 0.2% for 7 min. Note that addition of arabinose is not expected to impact the expression of PhoB-regulated genes. RNA was isolated using a modified hot-phenol method, as previously described (76). Samples were treated with Turbo DNase (Ambion) to remove genomic DNA, rRNA was removed using the Ribo-Zero rRNA removal kit for Gram-negative bacteria (Epicentre/Illumina), and libraries were prepared with the ScriptSeq Complete kit for bacteria (Epicentre/Illumina) (76). Libraries were sequenced on a HiSeq 2000 (Illumina) by the University at Buffalo Next-Generation Sequencing Core Facility. RNA-seq data were aligned to the E. coli MG1655 genome (GenBank accession no. NC_000913.3) using BWA for Illumina (v0.5.9-r16) (78) on Galaxy (https://usegalaxy.org) (79). Read counting, normalization, and differential expression analysis were performed in R using GenomicAlignments (v1.28) summarizeOverlaps (80) and DEseq2 (v1.32; betaPrior = FALSE) (81).

### PhoB motif discovery and analysis.

Sequences of 100 bp surrounding PhoB ChIP-seq peaks were extracted and analyzed using MEME (version 5.1.0; default parameters) (82, 83). The position of the inferred motif relative to ChIP-seq peak centers was analyzed using CentriMo (version 5.1.0; default parameters) (84) through the MEME-ChIP tool (85).

To determine whether the nucleotide content of the PhoB binding site motif contributes to the association of PhoB binding sites with H-NS-bound regions, we first scrambled each PhoB binding site individually using a custom Python script. We then compiled the scrambled sites into a PWM and searched the E. coli MG1655 genome (GenBank accession no. NC_000913.3) for the top 1,000 matches to this PWM using FIMO (version 5.1.0; default parameters) (86).

### Analysis of PhoB binding site conservation.

Binding site conservation analysis was performed as described previously (71). Protein sequences were aligned using Clustal Omega (87) and visualized using MView (88). The genomes analyzed were those of Arsenophonus nasoniae DSM 15247, *Brenneria* sp. EniD312, Cedecea davisae DSM 4568, Citrobacter rodentium ICC168, Cronobacter sakazakii ATCC BAA-894, Dickeya dadantii 3937, Edwardsiella tarda EIB202, Enterobacter cloacae subsp. *cloacae* ATCC 13047, Erwinia amylovora ATCC 49946, Escherichia coli strain K-12 substrain MG1655, Hafnia alvei ATCC 51873, Klebsiella pneumoniae KCTC 2242, Leminorella grimontii ATCC 33999 = DSM 5078, Morganella morganii subsp. *morganii* KT, Pantoea agglomerans 299R, Pectobacterium atrosepticum SCRI1043, Photorhabdus asymbiotica, Plesiomonas shigelloides 302-73, Proteus mirabilis HI4320, Providencia stuartii MRSN 2154, Pseudomonas aeruginosa PAO1, *Rahnella* sp. Y9602, Raoultella ornithinolytica B6, Salmonella enterica strain P125109, Serratia marcescens FGI94, Vibrio cholerae M66-2, Xenorhabdus bovienii SS-2004, Yersinia pestis KIM 10, and Yokenella regensburgei ATCC 43003.

### Data availability.

ChIP-seq data are available at EBI ArrayExpress under accession number E-MTAB-9293. RNA-seq data are available at EBI ArrayExpress under accession number E-MTAB-9591.
