# Supplementary material for: Genome-Wide Mapping of the Escherichia coli PhoB Regulon Reveals Many Transcriptionally Inert, Intragenic Binding Sites
Source: mBio. 2023 Apr 17;14(3):e02535-22. doi: 10.1128/mbio.02535-22 (PMC10294691; doi:10.1128/mbio.02535-22)
Supplement: FIG S1 [file mbio.02535-22-s0001.pdf]

	cov	id	
Escherichia	100.0%	100.0%	----------MARRILVVEDEAPIREMVCFVLEQNGFQPVEAEDYDSAVNQLNEPWPDLILLDWMLPGGSGIQFIKHLKR
Enterobacter	100.0%	96.5%	----------MARRILVVEDEAPIREMVCFVLEQNGFQPVEAEDYDSAVNQLNEPWPDLILLDWMLPGGSGLQFIKHLKR
Citrobacter	100.0%	95.6%	----------MARRILVVEDEAAIREMVCFVLEQNGFQPVEAEDYDTAVNQLNEPWPDLILLDWMLPGGSGLQFIKHLKR
Yokenella	100.0%	95.6%	----------MARRILVVEDEAPIREMVCFVLEQNGFQPVEAEDYDSAVNQLNEPWPDLILLDWMLPGGSGLQFIKHIKR
Salmonella	100.0%	95.6%	----------MARRILVVEDEAPIREMVCFVLEQNGFQPVEAEDYDSAVNKLNEPWPDLILLDWMLPGGSGLQFIKHLKR
Raoultella	100.0%	95.6%	----------MARRILVVEDEAPIREMVCFVLEQNGFQPVEAEDYDSAVNQLNEPWPDLILLDWMLPGGSGLQFIKLLKR
Klebsiella	100.0%	95.2%	----------MARRILVVEDEAPIREMVCFVLEQNGFQPVEAEDYDSAVNQLNEPWPDLILLDWMLPGGSGLQFIKLLKR
Cronobacter	100.0%	94.8%	----------MARRILVVEDEAPIREMVCFVLEQNGFQPVEAEDYDSAVNQLNEPWPDLILLDWMLPGGSGIQFIKHIKR
Cedecea	100.0%	92.1%	----------MARRILVVEDEAPIREMVSFVLEQNGFQSVEAEDYDSAVNLLIEPFPDLILLDWMLPGGSGIQFIKHLKR
Hafnia	100.0%	91.7%	----------MARRILVVEDEAPIREMVCFVLEQNGYQPVEAEDYDSAVNSLSEPYPDLVLLDWMLPGGSGIQFIKHMKR
Serratia	100.0%	91.3%	----------MARRILVVEDEAPIREMVCFVLEQNGYQPLEAEDYDSAVTRLSEPFPDLVLLDWMLPGGSGIQFIKHMKR
Pantoea	100.0%	90.4%	----------MAKRILVVEDEAPIREMLCFVLEQNDYQPIEAEDYDSAVGKLIEPWPDLILLDWMLPGGSGIQFIKHLKR
Brenneria	100.0%	89.5%	----------MARRILVVEDEAPIREMVCFVLEQNGYQPVEAEDYDSAVTQLSEPFPELVLLDWMLPGGSGLQFIKHMKR
Dickeya	100.0%	89.1%	----------MARRILVVEDEAPIREMVCFVLEQNGYQPVEAEDYDSAVTRLAEPYPELVLLDWMLPGGSGLQFIKHMKR
Rahnella	100.0%	89.1%	----------MARRILVVEDEAPIREMVCFVLEQNGYQAVEAEDFDSAIGQLVEPYPELILLDWMLPGGSGIQFIKHLKR
Pectobacterium	100.0%	89.1%	----------MAKRILVVEDEAPIREMVCFVLEQNGYQPVEAEDYDSAVTQLSEPFPELVLLDWMLPGGSGLQFIKHMKR
Erwinia	100.0%	87.8%	----------MAKRILVVEDEAPIREMLCFVLEQNDYQPIEAADYDSALSLLIEPWPDLILLDWMLPGGSGIQFIKHLKR
Yersinia	100.0%	86.2%	MTANILAGLMMARRILVVEDEAPIREMVCFVLEQNGYQPLEAEDYDSAVARLSEPFPDLVLLDWMLPGGSGIQFIKHMKR
Edwardsiella	100.0%	85.2%	----------MSIRILVVEDETPIRDMVSFVLEQNGYQPLEAESYDGALSQLCEPYPDLILLDWMLPGGSGIQLIKQLKR
Plesiomonas	100.0%	84.3%	----------MARRILVVEDEAPIRDMVCFVLEQKGYEPVEAEDYDAALSKMAEPYPDLILLDWMLPGGTGIQLIKHLKR
Providencia	100.0%	81.7%	----------MARRILVVEDEAPIREMVCFVLEQNGFQSIEADDYDSAIAQLVDPLPDLVLLDWMIPGGSGIQVIKHMKR
Xenorhabdus	100.0%	81.7%	----------MAKRILVVEDEVQIREMVCLVLEQNGYQTVEAEDYDVAVWRLSEPYPDLVLLDWMLPGGSGIQLIKQMKR
Photorhabdus	100.0%	81.2%	----------MTRRILVVEDETPIREMVCFVLEKNGYQPVEAEDYDSALACLSEPYPDLVLLDWMIPGGSGLQIIKQMKR
Morganella	100.0%	81.2%	----------MARRILVVEDEAPIREMVCFVLEQNGYQPIEADDYDAAIARLVEPFPDLVLLDWMIPGGSGIQVIKHMKR
Vibrio	100.0%	80.3%	----------MSRRILVVEDEAPIREMLCFVLEQKGYQAVEAEDYDSAMSKLAEPFPDLVLLDWMLPGGSGINLIKHMKR
Proteus	100.0%	79.0%	----------MARRILVVEDETAIREMICFVLEQNGFQPIEAEDYDTALSFLIDPYPDLVLLDWMIPGGSGIQVIKQMKR
Leminorella	100.0%	75.1%	----------MMKRILIVEDEAPIREMISLVLEQHDYQTVEAGDLASAQAQLKEPYPDLVLLDWMLPGGSGIQFIKSMKR
Arsenophonus	100.0%	74.8%	-----MIGSIMVRRILVVEDETAIREMVCFVLEQNGFQTVEADDYDSAIAQLIEPYPALILLDWMIPGGSGIQLIAHMKR
Pseudomonas	99.6%	61.3%	---------MVGKTILIVDDEAPIREMIAVALEMAGYECLEAENTQQAHAVIVDRKPDLILLDWMLPGTSGIELARRLKR

	cov	id	
Escherichia	100.0%	100.0%	ESMTRDIPVVMLTARGEEEDRVRGLETGADDYITKPFSPKELVARIKAVMRRISPMAVEEVIEMQGLSLDPTSHRVMAGE
Enterobacter	100.0%	96.5%	EAMTRDIPVVMLTARGEEEDRVRGLETGADDYITKPFSPKELVARIKAVMRRISPMAVEEVIEMQGLSLDPTSHRVMTGE
Citrobacter	100.0%	95.6%	EAMTRDIPVVMLTARGEEEDRVRGLETGADDYITKPFSPKELVARIKAVMRRISPMAVEEVIEMQGLSLDPTSHRVMTGD
Yokenella	100.0%	95.6%	EAMTRDIPVMMLTARGEEEDRVRGLETGADDYITKPFSPKELVARIKAVMRRISPMAVEEVIEMQGLSLDPTSHRVMTGE
Salmonella	100.0%	95.6%	EAMTRDIPVVMLTARGEEEDRVRGLETGADDYITKPFSPKELVARIKAVMRRISPMAVEEVIEMQGLSLDPGSHRVMTGD
Raoultella	100.0%	95.6%	EAMTRDIPVVMLTARGEEEDRVRGLETGADDYITKPFSPKELVARIKAVMRRISPMAVEEVIEMQGLSLDPSSHRVMTGE
Klebsiella	100.0%	95.2%	EAMTRDIPVVMLTARGEEEDRVRGLETGADDYITKPFSPKELVARIKAVMRRISPMAVEEVIEMQGLSLDPSSHRVMTGD
Cronobacter	100.0%	94.8%	EALTREIPVVMLTARGEEEDRVRGLETGADDYITKPFSPKELVARIKAVMRRISPMAVDEVIEMQGLSLDPSSHRVMTGE
Cedecea	100.0%	92.1%	EALTREIPVMMLTARGEEEDRVRGLEVGADDYITKPFSPKELVARIKAVMRRISPMAVEEVIEMQGLSLDPSSHRVMTGE
Hafnia	100.0%	91.7%	EALTREIPVMMLTARGEEEDRVRGLEVGADDYITKPFSPKELVARIKAVMRRISPMAVEEVIEVQGLSLDPTSHRVMANE
Serratia	100.0%	91.3%	EALTRDIPVMMLTARGEEEDRVRGLEVGADDYITKPFSPKELVARIKAVMRRISPMAVEEVIEMQGLSLDPSSHRVMANE
Pantoea	100.0%	90.4%	EAMTRDIPVMMLTARGEEEDRVRGLEVGADDYITKPFSPKELMARIKAVMRRISPMAVEEVIEMQGLSLDPSSHRVMSET
Brenneria	100.0%	89.5%	EALTRDIPVMMLTARGEEEDRVRGLEVGADDYITKPFSPKELVARIKAVMRRISPMAVEEVIEMRGLSLDPSSHRVTTEE
Dickeya	100.0%	89.1%	EALTRDIPVMMLTARGEEEDRVRGLEVGADDYITKPFSPKELVARIKAVMRRISPMAVEEVIEMRGLSLDPSSHRVTTEE
Rahnella	100.0%	89.1%	EALTREIPVMMLTARGEEEDRVRGLEVGADDYITKPFSPKELVARIKAVMRRISPMAVEEVIDMQGLSLDPSSHRVMANE
Pectobacterium	100.0%	89.1%	EALTRDIPVMMLTARGEEEDRVRGLEVGADDYITKPFSPKELVARIKAVMRRISPMAVEEVIEMRGLSLDPSSHRVTTEE
Erwinia	100.0%	87.8%	EAMTRDIPVMMLTARGEEEDRVRGLEVGADDYITKPFSPKELVARIKAVMRRIAPMAVEEVIEIQGLTLDPSSHRVMSGT
Yersinia	100.0%	86.2%	EALTRDIPVMMLTARGEEEDRVRGLEVGADDYITKPFSPKELVARIKAVMRRISPMAVEEVIEMQGLSLDPSSHRVMAND
Edwardsiella	100.0%	85.2%	EPATREIPVMMLTARGEEEDRVRGLEVGADDYITKPFSPKELVARIKAVMRRISPMALEETINLQGLSLDPVSHRVTAQD
Plesiomonas	100.0%	84.3%	EELTRNIPVVMLTARGEEEDRVRGLEVGADDYITKPFSPKELVARIKAVMRRIAPTSVEEVIDVQGLRLDPISHRVTAGE
Providencia	100.0%	81.7%	DSDTRDIPVMMLTARGEEEDRVKGLEVGADDYLIKPFSPKELVARVKAILRRISPMATEDIIEMNGLTLDPTSHRVSSND
Xenorhabdus	100.0%	81.7%	DSSTRDIPVMMVTARGEEEDRVRGLDVGADDYITKPFSPKELVARIKAVLRRVSPMSAEDIINMDGLILDSSSHRVSSQG
Photorhabdus	100.0%	81.2%	DNNVRDIPVMMLTARGEEEDRVRGLEIGADDYITKPFSPKELVARVKAVLRRISPMMIENLIEMDGLILDITSHRVTSQD
Morganella	100.0%	81.2%	DSQLREIPVMMLTARGEEEDRVKGLETGADDYLTKPFSPKELVARVKAILRRISPMAADDLIAMNGLTLDPASHRVTSNE
Vibrio	100.0%	80.3%	EEMTRNIPVVMLTARGEEEDKVRGLEVGADDYITKPFSPKELVARLKAVIRRVTPTALEDVIDVQGLKLDPVSHRVTAND
Proteus	100.0%	79.0%	ENNTRDIPIIMLTAKGEEEDKVKGLETGADDYVIKPFSPKELVARVKAILRRLSPMSAEDVIEFNGLSLDPVSHRVTSQD
Leminorella	100.0%	75.1%	EALTKDIPIIMLTARSEEDDRVQGLESGADDYVTKPFSPKELMARIKAVMRRASPMTEHEQLTFSGLVLDPSSHRVMADD
Arsenophonus	100.0%	74.8%	DQLTRNIPIMMLTARGEEEDRVRGLEIGADDYLTKPFSPKELIARVNAILRRVSAMASDNVIEMNGLKLDPASHRVMSDA
Pseudomonas	99.6%	61.3%	DELTVDIPIIMLTAKGEEDNKIQGLEVGADDYITKPFSPRELVARLKAVLRRTGPGDSEAPIEVGGLLLDPISHRVTIDG

```
               cov    id
Escherichia    100.0% 100.0%  EPLEMGPTEFKLLHFFMTHPERVYSREQLLNHVWGTNVYVEDRTVDVHIRRLRKALEPGGHDRMVQTVRGTGYRFSTRF
Enterobacter   100.0%  96.5%  NPLDMGPTEFKLLHFFMTHPERVYSREQLLNNVWGTNVYVEDRTVDVHIRRLRKALELSGHDRMVQTVRGTGYRFSTRF
Citrobacter    100.0%  95.6%  NPLDMGPTEFKLLHFFMTHPERVYSREQLLNHVWGTNVYVEDRTVDVHIRRLRKALETSGHDRMVQTVRGTGYRFSTRF
Yokenella      100.0%  95.6%  NPLDMGPTEFKLLHFFMTHPERVYSREQLLNHVWGTNVYVEDRTVDVHIRRLRKALEHSGHDRMVQTVRGTGYRFSARF
Salmonella     100.0%  95.6%  SPLDMGPTEFKLLHFFMTHPERVYSREQLLNHVWGTNVYVEDRTVDVHIRRLRKALEHSGHDRMVQTVRGTGYRFSTRF
Raoultella     100.0%  95.6%  SPLDMGPTEFKLLHFFMTHPERVYSREQLLNHVWGTNVYVEDRTVDVHIRRLRKALEHSGHDRMVQTVRGTGYRFSARF
Klebsiella     100.0%  95.2%  SPLDMGPTEFKLLHFFMTHPERVYSREQLLNHVWGTNVYVEDRTVDVHIRRLRKALEHSGHDRMVQTVRGTGYRFSARF
Cronobacter    100.0%  94.8%  NPLDMGPTEFKLLHFFMTHPERVYSREQLLNNVWGTNVYVEDRTVDVHIRRLRKALEQSGHDRMVQTVRGTGYRFSTRF
Cedecea        100.0%  92.1%  DPLDMGPTEFKLLHFFMTHPERVYSREQLLNNVWGTNVYVEDRTVDVHIRRLRKALETSGHDKMVQTVRGTGYRFSTRF
Hafnia         100.0%  91.7%  APLDMGPTEFKLLHFFMTHPERVYSREQLLNHVWGTNVYVEDRTVDVHIRRLRKALELSGHDKMVQTVRGTGYRFSARF
Serratia       100.0%  91.3%  QPLDMGPTEFKLLHFFMTHPERVYSREQLLNHVWGTNVYVEDRTVDVHIRRLRKALESSGHDKMVQTVRGTGYRFSTRY
Pantoea        100.0%  90.4%  TPLEMGPTEYKLLHFFMTHPERVYSREQLLNHVWGTNVYVEDRTVDVHIRRLRKALEVSGHDRMVQTVRGTGYRFSARY
Brenneria      100.0%  89.5%  LALDMGPTEFKLLHFFMTHPERVYSREQLLNHVWGTNVYVEDRTVDVHIRRLRKALETSGHDKMVQTVRGTGYRFSTRY
Dickeya        100.0%  89.1%  HALDMGPTEFKLLHFFMTHPERVYSREQLLNHVWGTNVYVEDRTVDVHIRRLRKALETSGHDKMVQTVRGTGYRFSTRY
Rahnella       100.0%  89.1%  QALDMGPTEFKLLHFFMTHSERVYSREQLLNNVWGTNVYVEDRTVDVHIRRLRKALETSGHDRMIQTVRGTGYRFSTRY
Pectobacterium 100.0%  89.1%  HALDMGPTEFKLLHFFMTHPERVYSREQLLNHVWGTNVYVEDRTVDVHIRRLRKALETSGHDKMVQTVRGTGYRFSTRY
Erwinia        100.0%  87.8%  QPLDMGPTEYKLLHFFMTHTERVYSREQLLNHIWGTNVYVEDRTVDVHIRRLRKALELSGHDRMVQTVRGTGYRFSARY
Yersinia       100.0%  86.2%  QALDMGPTEFKLLHFFMTHPERVYSREQLLNYVWGTNVYVEDRTVDVHIRRLRKALETDGHDKMVQTVRGTGYRFSTRY
Edwardsiella   100.0%  85.2%  DAVEMGPTEFKLLHFFMTHPERVYSREQLLNHVWGTNVYVEDRTVDVHIRRLRKALEGSGHDRMVQTVRGTGYRFSARF
Plesiomonas    100.0%  84.3%  QPLDMGPTEFKMLHFFMTHQERVYSREQLLNNVWGTNVYVEDRTVDVHIRRLRKALEGSGHDRLIQTVRGSGYRFSTRY
Providencia    100.0%  81.7%  TPIDMGPTEYKLLHFFMTHPERVYSREQLLNYVWGANVYVEDRTVDVHIRRLRKALEAEGHDKMVQTVRGTGYRFSVRY
Xenorhabdus    100.0%  81.7%  QNVEMGPTEFKLLHFFMTHPERVYSREQLLNYVWGTNVYVEDRTVDVHIRRLRKALEVGGHDRMVQTVRGTGYRFSTRY
Photorhabdus   100.0%  81.2%  QVLDMGPTEFKLLHFFMTHPERVYSREQLLNYVWGANVYVEDRTVDVHIRRLRKVLEVGGHDKMVQTVRGTGYRFSTRY
Morganella     100.0%  81.2%  NPLEMGPTEFKLLHFFMTHPERVYSREQLLNYVWGENVYVEDRTVDVHIRRLRKALEADGHDKMIQTVRGTGYRFSVRY
Vibrio         100.0%  80.3%  QPLDMGPTEFKMLHFFMTHQERVYSREQLLNNVWGTNVYVEDRTVDVHIRRLRKALEDAGHDKLIQTVRGAGYRFSTKA
Proteus        100.0%  79.0%  KPIDMGPTEFKLLHFFMTHPERVYSREQLLNYVWGTNVYVEDRTVDVHIRRLRKAIEQDGHDRMIQTVRGTGYRFSARF
Leminorella    100.0%  75.1%  VPLDMGPTEYKLLHFFMSHPERVYSREQLLNQVWGTNVYVEDRTVDVFIRRLRKTLEGSGYDKMLQTVRGSGYRFSTRF
Arsenophonus   100.0%  74.8%  TSIEMGPTEYKLLYFFMTHPERVYNREQLLNYVWGVNVYIEDRTVDVHIRRLRKALAVGGHEKMIQTVRGTGYRFSVRY
Pseudomonas     99.6%  61.3%  KPAEMGPTEYRLLQFFMTHQERAYTRGQLLDQVWGGNVYVEERTVDVHIRRLRKALG-EVYENLVQTVRGTGYRFSTKS
                                    **                 *       *      * **  ** ** *             * **  *
```

Figure S1. Alignment of PhoB protein sequences used for phylogenetic analysis of binding sites. PhoB protein sequences from 29 γ-proteobacterial species. Amino acids are color-coded by chemical property; amino acids are only colored if they are identical to the equivalent position in *E. coli*. Asterisks under the alignment indicate amino acids reported to contact DNA (32). The percent coverage (cov) and identity (id) relative to the *E. coli* sequence is indicated next to the genus name.
